# Deregulated miR-145 and miR-27b in Hutchinson-Gilford progeria syndrome: implications for adipogenesis

**DOI:** 10.18632/aging.206309

**Published:** 2025-08-27

**Authors:** Felix Quirin Fenzl, Eva-Maria Lederer, Louisa Brumma, Peter Krüger, Moritz Schroll, Frederic Wilming, Karima Djabali

**Affiliations:** 1 Epigenetics of Aging, Department of Dermatology and Allergy, TUM School of Medicine, Munich Institute of Biomedical Engineering (MIBE), Technical University of Munich (TUM), Garching 85748, Germany

**Keywords:** aging, Hutchinson-Gilford progeria syndrome (HGPS), progerin, microRNAs, adipogenesis

## Abstract

Hutchinson-Gilford progeria syndrome (HGPS) is a rare and fatal disorder that causes premature aging, affecting approximately one in 4–8 million births. Most cases result from a mutation in the lamin A/C (*LMNA*) gene, leading to the production of progerin, an aberrant lamin A variant that disrupts nuclear architecture and alters gene expression, including microRNA (miRNA) deregulation. This study aimed to investigate the molecular mechanisms underlying HGPS and aging using global miRNA sequencing to identify key deregulated miRNAs. Both miR-145 and miR-27b were significantly altered in HGPS. Functional experiments further revealed their crucial role in adipogenesis. Downregulation of these miRNAs in HGPS cells enhanced adipocyte differentiation, whereas their upregulation in control cells suppressed this process. These findings indicate that elevated levels of miR-145-5p and miR-27b-3p impair adipogenesis, providing mechanistic insights into HGPS pathophysiology and highlight new potential therapeutic avenues for both HGPS and metabolic disorders.

## INTRODUCTION

The Hutchinson-Gilford progeria syndrome (HGPS, OMIM #176670) is a rare autosomal dominant, and fatal genetic disorder, affecting approximately one in 4-8 million births worldwide, with equal prevalence across sexes and races [1, 2]. At birth, children with HGPS appear phenotypically normal, but within the first year, characteristic features emerge, including growth impairment, failure to thrive, lipodystrophy, alopecia, arthritis, and accelerated aging [2–5]. Patients typically succumb to severe atherosclerosis by the age of 14.6 years [6].

HGPS is most exclusively (18 of 20 classical cases) caused by a *de novo* point mutation in the lamin A/C (*LMNA*) gene (c.1824C>T, p.G608G) [7]. Lamin A, a key structural component of the nuclear lamina, plays a crucial role in maintaining nuclear integrity and genome organization [8, 9]. In normal cells, prelamin A, the precursor of lamin A undergoes post-transcriptional modifications, including farnesylation of the C-terminal cysteine in the CAAX motif (where C is cysteine, A is an aliphatic amino acid, and X is any amino acid), proteolytic cleavage of the AAX residues, carboxyl-methylation of the farnesylated cysteine, and finally removal of 14 terminal amino acids by a zinc metalloenzyme STE24 (ZMPSTE24) [10–12]. However, in HGPS, the G608G mutation creates a cryptic splice site in exon 11, leading to the loss of the ZMPSTE24 cleavage site and the production of progerin, a permanently farnesylated prelamin A variant. The accumulation of progerin disrupts the nuclear architecture, resulting in nuclear blebbing and cellular dysfunction, including telomere shortening, impaired DNA repair, mitochondrial dysfunction, oxidative stress, and premature cellular senescence [13–15].

Current therapeutic strategies for HGPS focus on correcting the mutation, reducing progerin levels, or mitigating its downstream effects [16]. One promising approach targets the overactivated Janus kinase-signal transducer and activator of transcription (JAK-STAT) signaling pathway, which contribute to progerin induced inflammation and cellular dysfunction [17, 18]. Lonafarnib, a farnesyltransferase inhibitor (FTI) and the only US Food and Drug Administration-approved treatment for HGPS, reduces progerin toxicity by preventing prelamin A farnesylation, thereby improving nuclear architecture, cardiovascular function, and extending lifespan to approximately 17-19.5 years [19–23].

Despite these advancements, a crucial yet understudied aspect of HGPS is the disruption of adipose tissue homeostasis [24]. Adipose tissue, essential for energy storage, thermogenesis, and metabolic regulation, also secretes adipokines and cytokines [25, 26]. In patients with HGPS, adipose tissue deficiency contributes to metabolic dysregulation and cardiovascular complications [26, 27]. Defective adipogenesis, characterized by impaired adipocyte differentiation, disrupted lipid droplet formation, and defective triglyceride transport, underlies lipodystrophy in HGPS [28]. Key transcription factors, including peroxisome proliferator-activated receptor gamma (PPARγ) and CCAAT/enhancer-binding protein alpha (C/EBPα) [29], regulate the expression of essential genes, such as fatty acid binding protein 4 (FABP4) and lipoprotein lipase (LPL) both of which are important for mature adipocytes function [30, 31].

Dysregulated microRNAs (miRNAs) play a crucial role in adipogenesis [32]. Progerin-induced heterochromatin loss activates normally silent chromatin regions, leading to transcriptional dysregulation, including alteration in major regulatory factors [33, 34]. Additionally, aberrant expression of RNA polymerase II further disrupts miRNA homeostasis, as this enzyme transcribes primary miRNA (pri-miRNA) transcripts [35, 36]. MiRNAs, the small non-coding RNAs ranging from 19 to 25 nucleotides in length, act as post-transcriptional regulators of gene expression [37].

This study aimed to investigate the molecular mechanisms underlying HGPS and aging using global miRNA sequencing. Cellular aging was assessed based on replicative senescence, characterized by a progressive decline in proliferative capacity, and quantified as the proportion of senescent cells relative to the Hayflick limit [38, 39]. Specifically, we examined miR-145-5p and miR-27b-3p, which are dysregulated in HGPS, to elucidate their roles in the adipogenic pathway. Our findings provide critical insights into disease pathogenesis and highlight potential therapeutic targets for mitigating metabolic complications associated with HGPS.

## RESULTS

### Genome-wide miRNA sequencing reveals distinct miRNA signatures in normal and premature aging

**Figure 1:**
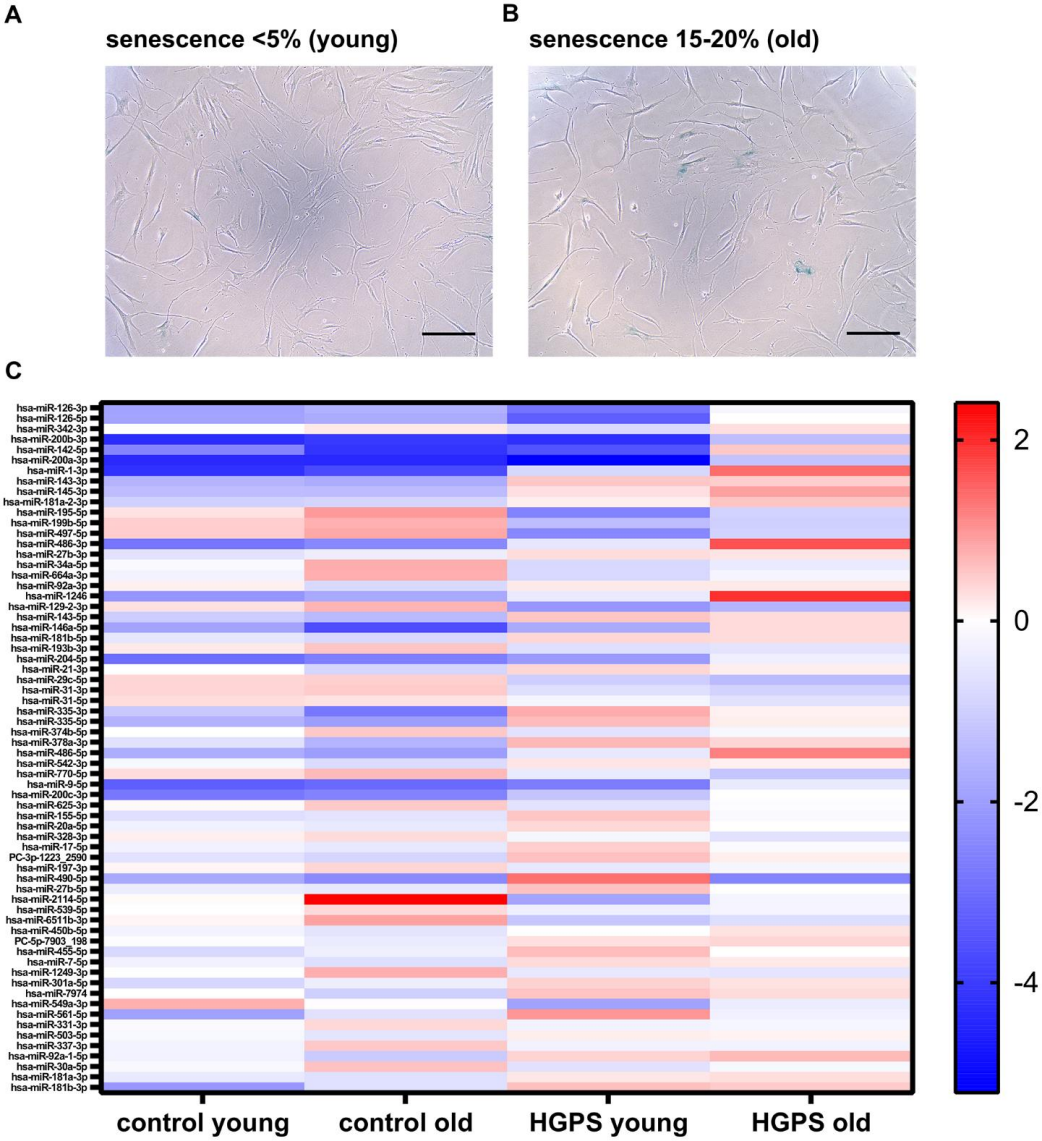
**Genome-wide sequencing of miRNAs in control and HGPS fibroblasts across cellular ages.** (**A**) Representative SA-β-galactosidase-stained cells (10x magnification; scale bar 100 μm) show control fibroblasts at young passages with < 5% senescence and (**B**) old passages with 15-20% senescence. (**C**) A total of 66 significantly deregulated miRNAs were identified across six comparisons (control old vs. control young; HGPS old vs. HGPS young; young HGPS vs. young control; old HGPS vs. old control; old HGPS vs. young control; young HGPS vs. old control). Genome-wide miRNA profiles were generated from control cell strains (GM01651, GM01652, GM03349) and HGPS cell strains (HGADFN003, HGADFN127, HGADFN178) at <5% and 15-20% senescence. (Also see Supplementary Table 1).

To elucidate the molecular mechanisms underlying HGPS and normal aging, we performed global miRNA sequencing to identify differentially expressed miRNAs associated with both normal and premature aging. We conducted single-end sequencing (50 bp reads) on control cell strains (GM01651, GM01652, GM03349) and HGPS cell strains (HGADFN003, HGADFN127, HGADFN178), all carrying a heterozygous c.1824C>T (p.Gly608Gly) mutation in *LMNA* exon 11. This analysis was performed on both young (<5% senescence, Figure 1A) and older passages (15–20% senescence, Figure 1B).

A total of 66 significantly deregulated miRNAs were identified (Figure 1C) across six major comparisons: normal aging (control old vs. control young); premature aging (HGPS old vs. HGPS young); early molecular changes (young HGPS vs. young control); advanced cellular changes (old HGPS vs. old control); aging and disease progression (old HGPS vs. young control); premature versus normal aging (young HGPS vs. old control). Several miRNAs exhibited differential expression across multiple comparisons, suggesting their potential regulatory roles in both normal and accelerated aging (see Supplementary Table 1).

Additionally, we identified 37 differentially expressed miRNAs in four key comparisons (Table 1): normal aging (control old vs. control young); premature aging (HGPS old vs. HGPS young); early molecular changes (young HGPS vs. young control); and late-stages of cellular aging (old HGPS vs. old control). These differentially expressed miRNAs were further analyzed using Ingenuity Pathway Analysis (IPA) software, (https://apps.ingenuity.com) to identify experimentally validated gene targets and associated canonical pathways (Supplementary Figure 1).

**Table 1:** Differentially expressed miRNAs between normal and HGPS fibroblasts across cellular ages.

		**Control old vs. control young**			**HGPS old vs. HGPS young**			**Young HGPS vs. young control**			**Old HGPS vs. old control**		
		**log2FC**	**p-value**	**q-value**	**log2FC**	**p-value**	**q-value**	**log2FC**	**p-value**	**q-value**	**log2FC**	**p-value**	**q-value**
1	hsa-miR-126-3p	0,6	5,2E-01	1,0E+00	3,4	1,27E-04	7,32E-03	-0,8	3,52E-01	9,88E-01	2,0	2,32E-02	3,13E-01
2	hsa-miR-126-5p	0,3	7,0E-01	1,0E+00	3,9	6,64E-06	4,85E-04	-1,5	8,15E-02	8,20E-01	2,1	1,50E-02	2,44E-01
3	hsa-miR-342-3p	0,2	3,7E-01	1,0E+00	1,0	1,77E-04	9,53E-03	-0,7	7,98E-03	2,30E-01	0,1	8,05E-01	9,72E-01
4	hsa-miR-200b-3p	0,5	7,6E-01	1,0E+00	5,9	6,79E-04	3,16E-02	-1,3	4,67E-01	9,88E-01	4,1	1,70E-02	2,60E-01
5	hsa-miR-142-5p	-3,2	2,1E-02	6,1E-01	7,0	8,38E-07	7,80E-05	-3,5	1,41E-02	3,16E-01	6,7	1,58E-06	2,02E-04
6	hsa-miR-200a-3p	0,4	7,2E-01	1,0E+00	5,8	1,00E-06	8,44E-05	-0,9	4,35E-01	9,88E-01	4,4	1,29E-04	6,01E-03
7	hsa-miR-1-3p	-0,2	8,9E-01	1,0E+00	1,6	2,44E-01	9,99E-01	5,6	1,16E-04	8,74E-03	7,4	6,82E-07	9,97E-05
8	hsa-miR-143-3p	-0,1	8,3E-01	1,0E+00	-0,1	8,95E-01	9,99E-01	2,1	9,63E-04	5,79E-02	2,2	7,20E-04	2,38E-02
9	hsa-miR-145-3p	0,0	9,6E-01	1,0E+00	0,5	2,56E-01	9,99E-01	1,7	9,60E-05	8,74E-03	2,3	3,67E-07	7,50E-05
10	hsa-miR-181a-2-3p	0,1	7,2E-01	1,0E+00	0,4	2,28E-01	9,99E-01	1,1	8,59E-04	5,49E-02	1,4	3,16E-05	1,70E-03
11	hsa-miR-195-5p	0,7	2,2E-01	1,0E+00	1,5	1,01E-02	3,44E-01	-2,6	1,16E-05	1,69E-03	-1,8	2,63E-03	6,11E-02
12	hsa-miR-199b-5p	0,4	3,0E-01	1,0E+00	0,4	2,45E-01	9,99E-01	-1,8	1,48E-07	7,56E-05	-1,7	3,05E-07	7,50E-05
13	hsa-miR-497-5p	0,4	4,5E-01	1,0E+00	1,2	3,70E-02	8,64E-01	-2,6	6,06E-06	1,24E-03	-1,8	2,03E-03	5,19E-02
14	hsa-miR-486-3p	3,1	4,0E-02	8,5E-01	1,1	4,11E-01	9,99E-01	4,6	1,91E-03	9,30E-02	2,6	5,54E-02	5,11E-01
15	hsa-miR-27b-3p	0,2	4,1E-01	1,0E+00	-0,1	6,82E-01	9,99E-01	0,9	1,26E-03	7,18E-02	0,6	4,66E-02	4,56E-01
16	hsa-miR-34a-5p	0,9	1,16E-03	7,44E-02	0,3	2,49E-01	9,99E-01	-0,6	2,45E-02	3,92E-01	-1,2	1,61E-05	1,17E-03
17	hsa-miR-664a-3p	1,0	4,49E-04	3,53E-02	0,6	6,46E-02	9,99E-01	-0,5	9,07E-02	8,74E-01	-1,0	2,18E-03	5,30E-02
18	hsa-miR-92a-3p	-0,9	6,38E-05	7,08E-03	0,0	8,86E-01	9,99E-01	0,1	6,92E-01	9,88E-01	0,9	2,48E-05	1,49E-03
19	hsa-miR-1246	-1,1	4,31E-01	1,00E+00	1,1	4,02E-01	9,99E-01	3,2	1,56E-02	3,21E-01	5,4	1,45E-04	6,46E-03
20	hsa-miR-129-2-3p	0,7	3,95E-01	1,00E+00	0,1	8,92E-01	9,99E-01	-1,9	1,63E-02	3,21E-01	-2,5	2,29E-03	5,46E-02
21	hsa-miR-143-5p	-0,3	6,44E-01	1,00E+00	-0,1	9,23E-01	9,99E-01	1,6	6,17E-03	2,04E-01	1,8	1,93E-03	5,05E-02
22	hsa-miR-146a-5p	-1,5	7,19E-02	1,00E+00	1,6	6,67E-02	9,99E-01	0,7	4,25E-01	9,88E-01	3,8	1,02E-05	8,68E-04
23	hsa-miR-181b-5p	-0,2	5,25E-01	1,00E+00	0,0	9,15E-01	9,99E-01	0,9	8,10E-03	2,30E-01	1,1	1,57E-03	4,33E-02
24	hsa-miR-193b-3p	0,4	1,67E-01	1,00E+00	0,1	7,41E-01	9,99E-01	-0,8	4,96E-03	1,85E-01	-1,2	1,26E-04	6,01E-03
25	hsa-miR-204-5p	0,3	9,40E-01	1,00E+00	2,9	3,45E-01	9,99E-01	6,8	3,64E-02	4,83E-01	9,5	3,55E-03	7,73E-02
26	hsa-miR-21-3p	-0,8	1,09E-02	4,44E-01	-0,2	4,68E-01	9,99E-01	0,4	1,75E-01	9,88E-01	1,0	1,53E-03	4,33E-02
27	hsa-miR-29c-5p	0,1	8,41E-01	1,00E+00	-1,0	2,34E-01	9,99E-01	-1,2	7,84E-02	8,10E-01	-2,3	3,18E-03	7,09E-02
28	hsa-miR-31-3p	0,1	7,09E-01	1,00E+00	-0,2	6,10E-01	9,99E-01	-1,1	4,72E-03	1,85E-01	-1,4	2,48E-04	9,78E-03
29	hsa-miR-31-5p	0,0	8,84E-01	1,00E+00	-0,4	1,29E-01	9,99E-01	-0,5	4,25E-02	5,30E-01	-0,9	6,92E-04	2,36E-02
30	hsa-miR-335-3p	-1,6	3,66E-02	8,31E-01	-0,6	4,15E-01	9,99E-01	1,8	1,66E-02	3,21E-01	2,7	2,93E-04	1,11E-02
31	hsa-miR-335-5p	-0,6	3,91E-01	1,00E+00	-0,3	7,27E-01	9,99E-01	1,9	8,98E-03	2,30E-01	2,3	2,11E-03	5,27E-02
32	hsa-miR-374b-5p	0,6	2,08E-02	6,13E-01	0,4	1,24E-01	9,99E-01	-0,5	2,55E-02	4,01E-01	-0,7	4,45E-03	9,48E-02
33	hsa-miR-378a-3p	-1,0	3,00E-02	7,70E-01	-0,3	4,49E-01	9,99E-01	1,2	4,49E-03	1,84E-01	1,9	3,87E-05	1,98E-03
34	hsa-miR-486-5p	-0,2	7,51E-01	1,00E+00	1,7	1,17E-02	3,76E-01	1,1	9,59E-02	9,00E-01	3,0	6,98E-06	6,49E-04
35	hsa-miR-542-3p	-0,5	4,86E-02	9,38E-01	-0,1	5,98E-01	9,99E-01	0,4	1,27E-01	9,88E-01	0,8	3,19E-03	7,09E-02
36	hsa-miR-770-5p	0,3	5,22E-01	1,00E+00	-1,1	8,48E-02	9,99E-01	-0,7	2,02E-01	9,88E-01	-2,2	6,77E-04	2,36E-02
37	hsa-miR-9-5p	0,4	8,65E-01	1,00E+00	5,9	2,96E-03	1,12E-01	0,8	6,97E-01	9,88E-01	6,4	1,44E-03	4,33E-02

A total of 37 significantly differentially expressed miRNAs were identified in four main comparisons: control old vs. control young; HGPS old vs. HGPS young; young HGPS vs. young control; old HGPS vs. old control; including log2FoldChange (log2FC), p-value, and q-value, with distinct miRNA sets (bold, respective color). Genome-wide miRNA profiles were identified from control (GM01651, GM01652, GM03349) and HGPS cell strains (HGADFN003, HGADFN127, HGADFN178) at <5% and 15-20% senescence. A comprehensive list of all 66 significantly deregulated miRNAs across six comparisons is detailed in Supplementary Table 1.

### miRNA signature in normal and HGPS aging

On comparing late passages (old) control cells with 15 to 20% senescence to early-passages (young) control cells with <5% senescence, we identified three differentially expressed miRNAs: miR-34a-5p, miR-664a-3p, and miR-92a-3p (Table 1). Using the IPA microRNA target filter tool, we identified experimentally validated targets for miR-34a-5p and miR-92a-3p (Supplementary Figure 1A, 1B). miR-34a-5p (log 2-fold change [log2FC]: 0.9) was upregulated in old control cells and was associated with cellular senescence, apoptosis, and tissue regeneration, suggesting a pro-aging role (Supplementary Table 2). In contrast, miR-92a-3p (log2FC: -0.9) was downregulated in old control cells and was linked to cell cycle regulation, apoptosis, DNA repair, and inflammation, indicating a protective function in aging (Supplementary Table 2).

On comparing old HGPS cells (15–20% senescence) to young HGPS cells (<5% senescence), we identified six differentially expressed miRNAs: miR-126-3p, miR-126-5p, miR-342-3p, miR-200a-3p, miR-200b-3p, and miR-142-5p (Table 1). Pathway analysis of experimentally validated targets using Ingenuity canonical pathways revealed two distinct miRNA clusters associated with aging. The first cluster, consisting of miR-200a and miR-200b, was linked to epithelial-mesenchymal transition (EMT), cellular senescence, and fibrosis [40] (Supplementary Figure 1C). The second cluster, comprising miR-126a-3p and miRNA-126a-5p, was associated with vascular homeostasis and identified as a key regulator of premature aging [41, 42] (Supplementary Figure 1D and Supplementary Table 2).

### miRNA signature of early molecular changes in HGPS

A comparison between young HGPS cells and young control cells (both <5% senescence) identified nine differentially expressed miRNAs (Table 1). Among them, miR-1-3p (log2FC: 5.6) and miR-143-3p (log2FC: 2.1) were significantly upregulated, whereas miR-199a-5p (log2FC: -1.8), miR-27b-3p (log2FC: 0.9), miR-497-5p (log2FC: -2.6), and miR-195-5p (log2FC: -2.6) were downregulated (Table 1). Pathway analysis indicated that these miRNAs are involved in apoptosis, growth factor signaling, epigenetic regulation, and cell cycle control. Disruptions in these critical pathways likely contribute to the accelerated cellular aging in HGPS, highlighting the early onset of molecular alterations in the disease (Supplementary Figure 1E and Supplementary Table 2).

### miRNA signature of premature aging in HGPS

A comparison between late-passage (old) HGPS and late-passage (old) control cells, both exhibiting 15–20% senescence, identified 31 differentially expressed miRNAs (Table 1). Analysis of shared and experimentally validated targets identified distinct miRNA clusters associated with apoptosis, cell cycle progression, and tissue homeostasis. These findings suggest that dysregulation of these miRNAs contributes to the accelerated aging phenotype observed in HGPS (Supplementary Figure 1F and Supplementary Table 2).

### Consistent miRNA alterations in HGPS

A comparative analysis between young (early-) and old (late-passage) HGPS cells showed consistent dysregulation. Specifically, miR-1-3p, miR-143-3p, miR-145-3p, and miR-181a-2-3p were consistently upregulated, whereas miR-195-5p, 199b-5p, and miR-497-5p were downregulated relative to controls (Table 1). The miR-143/145 and the miR-195/497 clusters exhibited coordinated expression patterns [43], suggesting a shared regulatory mechanism that may amplify their effects on gene expression and cellular processes [44]. The miR-195-5p and miR-497-5p, which share identical seed sequences and are clustered on chromosome 17 [45], were markedly downregulated in young HGPS cells (log2FC: -2.6) and moderately downregulated in older HGPS cells (log2FC: -1.8) (Table 1). This suppression may be attributed to elevated NF-κB activity, which is known to repress the miR-195/497 expression [46]. This finding aligns with previous reports of heightened NF-kB activation in HGPS cells [47, 48].

Conversely, miR-143 and miR-145, co-transcribed from chromosome 5 [49], were significantly upregulated in both young (miR-143 log2FC: 2.1; miR-145 log2FC: 1.7) and old HGPS cell (miR-143 log2FC: 2.2; miR-145 log2FC: 2.3), (Table 1). This cluster is involved in the regulation of cell proliferation, apoptosis, and differentiation [50, 51] and is highly expressed in vascular smooth muscle cells [49], where it is essential for maintaining vascular function [52, 53]. Dysregulation of miR-143/145 may contribute to the vascular pathology of HGPS, including atherosclerosis, a leading cause of mortality in HGPS [52, 53]. Additionally, given its involvement in insulin signaling, the deregulation of the miR-143/145 cluster may contribute to metabolism dysfunctions and impaired adipogenesis observed in HGPS [54].

### miRNAs drive early HGPS changes

Although several miRNAs exhibit overlapping changes in HGPS, miR-486-3p (log2FC: 4.6) and miR-27b-3p (log2FC: 0.9) play distinct roles in early disease pathogenesis (Table 1). miRNA-486-3p, which targets the androgen receptor (AR), is markedly upregulated and has been implicated in promoting premature senescence in HGPS [55, 56] (Supplementary Figure 1G). In contrast, miR-27b-3p influences cellular metabolism by regulating prohibitin (PHB1), peroxisome proliferator-activated receptor gamma (PPARγ), retinoid X receptor alpha (RXRA), and fatty acid synthase (FASN), highlighting it as a strong candidate whose deregulation in HGPS may contribute to impaired adipogenesis [57–60] (Supplementary Figure 1H).

### Adipocyte differentiation is impaired in HGPS

**Figure 2:**
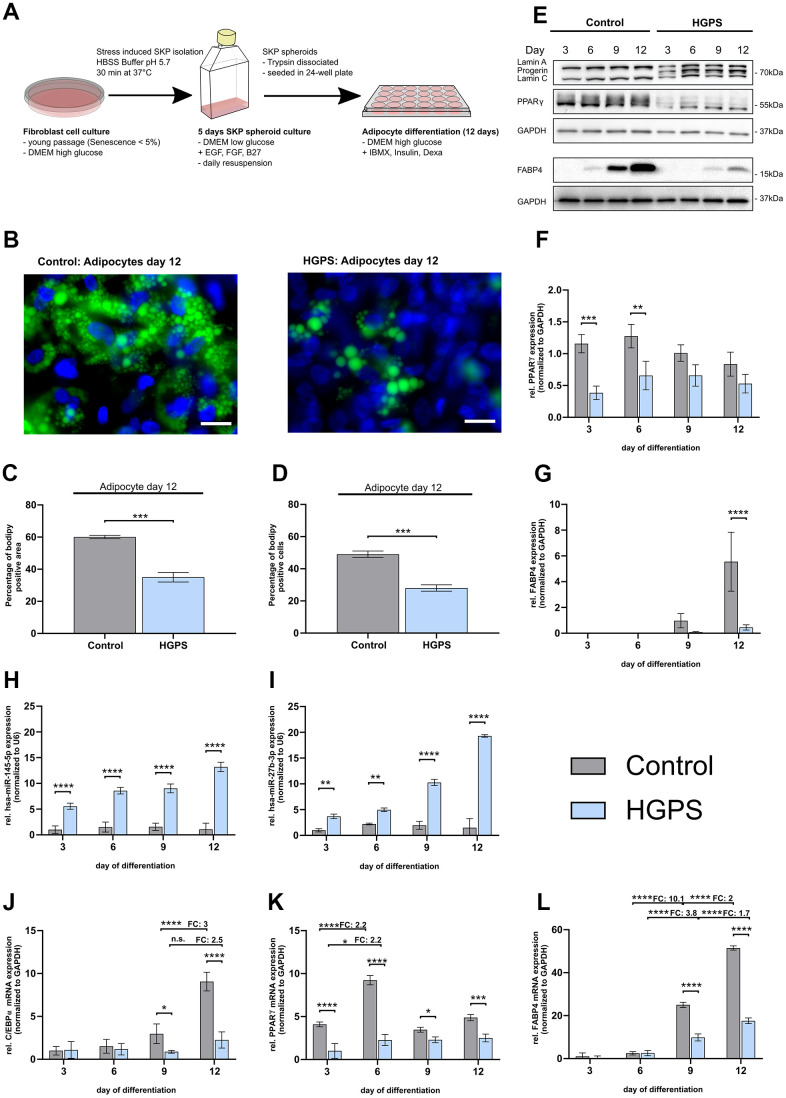
**Impaired adipocyte differentiation in HGPS compared to control cells.** Control cell strains: GM05565, GM05757, and GM01651; HGPS cell strains: HGADFN003, HGADFN127, and HGADFN164; (**A**) Schematic outline of adipocyte differentiation protocol; (**B**) Bodipy staining of lipid vesicles (green) in control and HGPS adipocytes on day 12 (40x magnification; scale bar 20 μm) with DAPI counterstaining. (**C**) Quantification of the Bodipy-positive area, normalized to the DAPI signal area. (**D**) Quantification of the percentage of Bodipy-positive cells normalized to DAPI positive cells. (**E**) Representative western blot of Lamin A/C including progerin, PPARγ and FABP4 at day 3, 6, 9 and 12 of adipogenesis (for uncropped and unprocessed images, see Supplementary Figure 3). Quantification of PPARγ (**F**) and FABP4 (**G**) protein level normalized to GAPDH. qPCR analysis of miR-145-5p (**H**) and miR-27b-3p (**I**) normalized to U6 and mRNA levels of C/EBPα (**J**), PPARγ (**K**), and FABP4 (**L**) normalized to GAPDH. (**J**–**L**) Values are presented as the mean ± SD (n=3); p > 0.05; * p < 0.05; ** p < 0.01; *** p < 0.001; **** p < 0.0001; (**B**, **C**) unpaired t-test; (**E**–**I**) two-way ANOVA with Sidak’s multiple comparisons test.

Genome-wide miRNA sequencing identified an upregulation of miR-145 and miR-27b in HGPS fibroblasts, with downstream target analysis indicating their potential contribution to impaired adipogenesis (Table 1). To investigate this, we developed an *ex vivo* adipogenesis model using skin-derived precursor (SKP) cells isolated from primary human fibroblast cultures using the low-pH stress method [61]. To minimize the confounding effects of cellular senescence, we used early-passage cultures (<5% senescence) (Figure 1A and Supplementary Table 3) [62]. Dissociated SKP spheroids were cultured in adipocyte differentiation medium for 12 days, following established protocols [63] (Figure 2A). SKPs differentiated under this protocol first acquire a preadipocyte phenotype, expressing canonical white adipogenic transcription factors such as PPARγ, C/EBPα, and FABP4, as shown by Budel et al. [63]. Although full maturation into unilocular lipid-droplet-containing white adipocytes is rarely achieved *in vitro*, the protocol is widely used to model early white adipogenesis. Further investigation, including UCP1 profiling, would be required to formally exclude beige adipocyte features.

On day 12, Bodipy staining showed significantly reduced lipid droplet formation in HGPS cultures compared to controls, with only 28% of HGPS cells differentiating into adipocytes, compared to 49% of control cells (Figures 2B–2D). The Bodipy-positive area in HGPS cultures was 35%, nearly half of that observed in the control groups (60%), consistent with previous findings that indicate impaired adipocyte differentiation in HGPS [62, 63].

To further investigate the impaired adipogenesis in HGPS, we analyzed the expression of PPARγ, a key adipogenic transcription factor [64–68]. Western blotting showed consistently lower PPARγ expression in HGPS cells throughout differentiation (Figure 2E, 2F). Lamin A/C immunoblots showed a distinct progerin band in HGPS samples at days 3, 6, 9, and 12, with increasing intensity over time, confirming progressive progerin accumulation and its involvement in HGPS pathology (Figure 2E and Supplementary Figure 2). Moreover, the late adipogenic marker FABP4 [24] exhibited delayed and reduced expression in HGPS cells with significantly lower protein levels from day 9 to day 12, compared to controls (Figure 2G).

We further analyzed the expression patterns of miR-145-5p and miR-27b-3p and adipogenesis markers (C/EBPα, PPARγ, and FABP4) during the 12-day differentiation period (Figures 2H–2L). Notably, miR-145-5p and miR-27b-3p were significantly upregulated in HGPS cells as early as day 3, reaching 12-fold and 13-fold increases, respectively by day 12, whereas control cells showed no significant change (Figure 2H, 2I). This elevated miRNA expression corresponded with suppressed mRNA levels of key adipogenic markers. Specifically, C/EBPα mRNA expression was significantly lower in HGPS cells than in controls on days 9 and 12 (Figure 2J), whereas PPARγ mRNA levels increased similarly in both HGPS and control cells from day 3 to day 6 (2.2-fold), with consistently lower expression in HGPS cells throughout the differentiation (Figure 2K). The late adipogenesis marker FABP4 exhibited a strong upregulation in control cells at day 9 (10.1-fold compared to day 6) and day 12 (2-fold compared to day 9), whereas HGPS cells maintained significantly lower expression (Figure 2L). These findings confirm that the upregulation of miR-145-5p and miR-27b-3p negatively affects adipogenesis, providing a mechanistic link between miRNA dysregulation and the impaired adipogenic capacity observed in HGPS.

### Deregulated miR-145a-5p and miR-27b-3p in adipogenic differentiation of white adipose tissue (WAT) in Lmna^G609G/G609G^ mice

**Figure 3:**
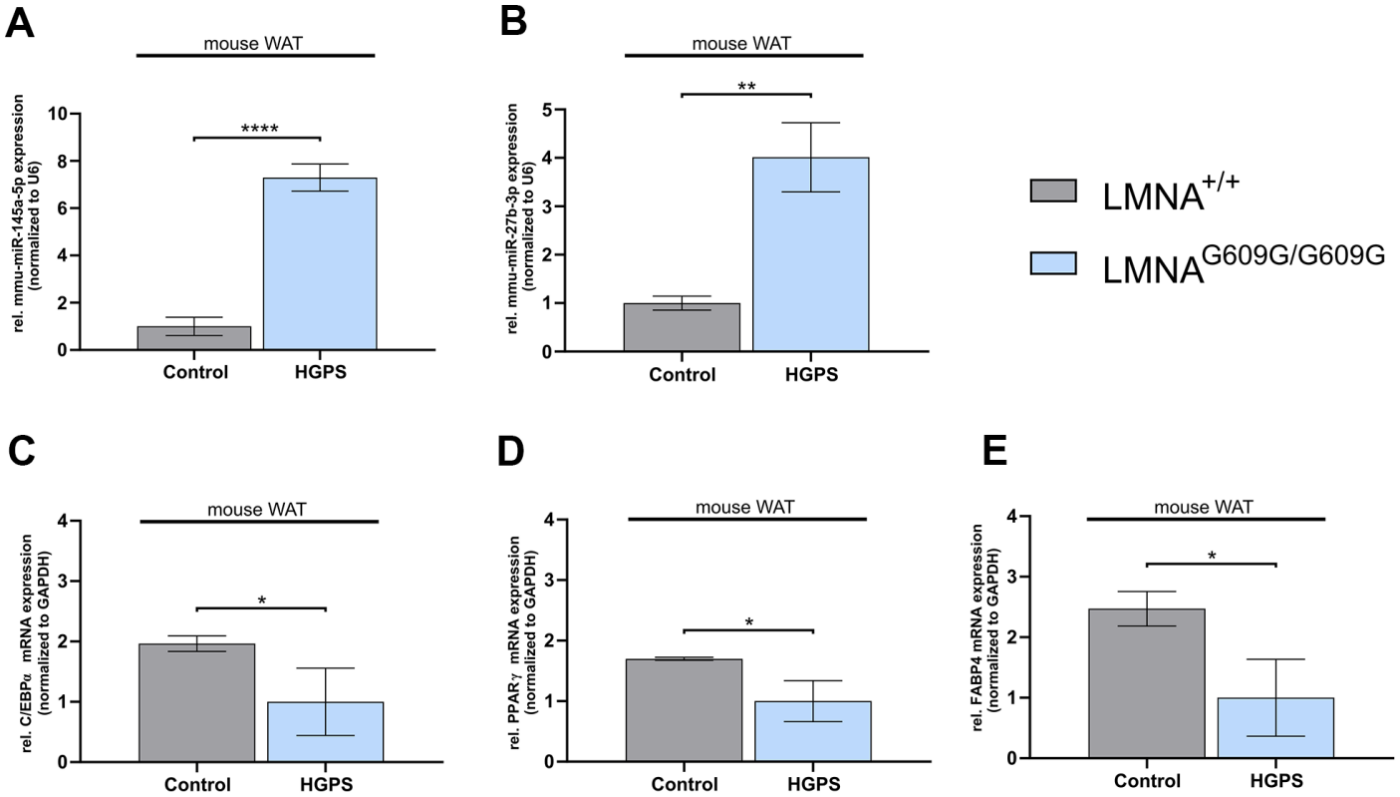
**Deregulated mmu-miR-145a-5p and mmu-miR-27b-3p levels, along with deregulated adipogenic differentiation genes in white adipose tissue (WAT) of Lmna^G609G/G609G^ mice.** (**A**) qPCR analysis of miR-145-5p and (**B**) miR-27b-3p, both normalized to U6. (**C**) mRNA levels of CCAAT/enhancer binding protein-alpha (C/EBPα), (**D**) peroxisome proliferation-activated receptor gamma PPARγ and (**E**) fatty acid binding protein 4 (FABP4), all normalized to GAPDH. (**A**–**E**) Values are presented as the mean ± SD (n=3); p > 0.05; * p < 0.05; ** p < 0.01; *** p < 0.001; **** p < 0.0001 (unpaired t-test).

Progerin expression in the HGPS mouse model is associated with lipodystrophic features and metabolic dysfunction, mirroring those observed in patients with HGPS [69, 70]. To determine whether elevated miR-145-5p and miR-27b-3p expression correlate with reduced expression of key adipogenic markers (C/EBPα, PPARγ, and FABP4), we analyzed WAT from Lmna^G609G/G609G^ mice and compared it to age-matched wild-type mice using qRT-PCR (Figure 3) [71].

Both miR-145a-5p and miR-27b-3p were significantly upregulated in the WAT of Lmna^G609G/G609G^ mice, exhibiting 7- and 4-fold increases, respectively (Figure 3A, 3B). This upregulation was accompanied by a marked downregulation of C/EBPα, PPARγ, and FABP4, in HGPS mice relative to wild-type controls (Figures 3C–3E). These findings suggest that miRNA dysregulation contributes to lipodystrophy in HGPS, indicating that miR-145-5p and miR-27b-3p modulation could act as potential therapeutic targets for restoring adipogenesis.

### miR-145-5p and miR-27b-3p inhibition enhances adipogenic differentiation in HGPS

**Figure 4:**
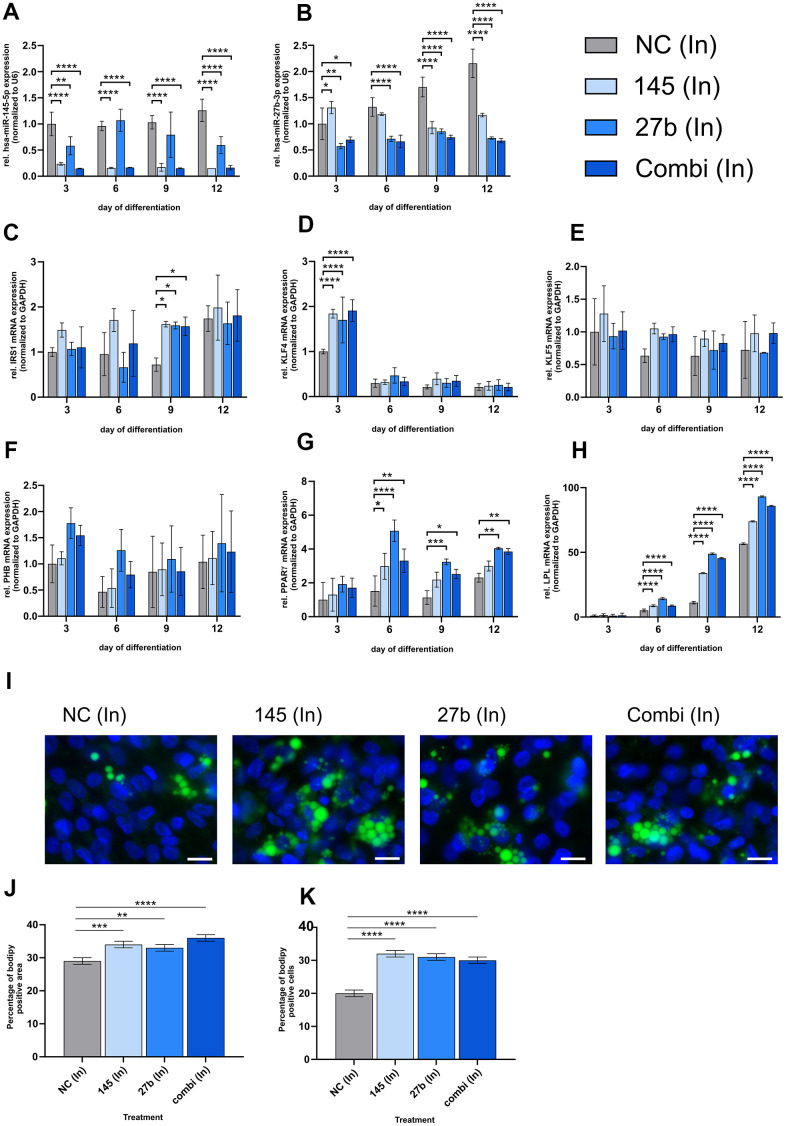
**miR-145-5p and miR-27b-3p inhibitors enhance adipogenic differentiation in HGPS cells.** HGPS cell strains (HGADFN003, HGADFN127, and HGADFN164) were transfected with 10 nM hsa-miR-145-5p and/or has-miR-27b-3p inhibitors on days 0, 3, 6, and 9 during SKP-to-adipocyte differentiation. The treatments groups include 10 nM negative control inhibitor (NC (In)), 10 nM hsa-miR-145-5p inhibitor (145 (In)), 10 nM hsa-miR-27b-3p inhibitor (27b (In)) and a combination of 10 nM hsa-miR-145-5p inhibitor and 10 nM hsa-miR-27b-3p inhibitor (Combi (In)); qPCR analysis was performed for miR-145-5p (**A**), and miR-27b-3p (**B**), both normalized to U6, as well as for IRS1 (**C**), KLF4 (**D**), KLF5 (**E**), PHB (**F**), PPARγ (**G**) and LPL (**H**) all normalized to GAPDH. (**I**) Bodipy staining (green) of lipid droplets on day 12 of differentiation with representative images (40x magnification; scale bar: 20 μm). DAPI was used as a counterstain. (**J**) Quantification of the percentage of Bodipy-positive area normalized to the total number of DAPI area. (**K**) Quantification of the percentage of Bodipy-positive cells normalized to the total number of DAPI-positive cells. (**A**–**H**) Values are presented as mean ± SD (n=3); p > 0.05; * p < 0.05; ** p < 0.01; *** p < 0.001; **** p < 0.0001; two-way ANOVA with Tukey’s multiple comparison test. (**J**, **K**) Values are presented as the mean ± SD (n=3); p > 0.05; * p < 0.05; ** p < 0.01; *** p < 0.001; **** p < 0.0001; Ordinary one-way ANOVA with Dunnett’s multiple comparison test.

To determine whether downregulating miR-145-5p and miR-27b-3p could improve adipogenic potential, we transfected HGPS SKPs with 10 nM miR-145-5p and/or miR-27b-3p inhibitors every 3 days during differentiation (Figure 4). Subsequently, we assessed miRNA levels and target mRNA expression using qRT-PCR (Figure 4A–4H).

Inhibitor transfection led to a significant reduction in miR-145-5p and miR-27b-3p levels throughout differentiation (Figures 4A, 4B). miR-145-5p inhibition alone or in combination with miR-27b-3p demonstrated greater suppression than miR-27b-3p inhibition alone, suggesting a potential interaction between the two miRNAs.

Insulin receptor substrate 1 (IRS1) levels were significantly elevated following miR-145-5p inhibition, with the highest increase on day 9 (Figure 4C). KLF4, an early adipogenesis marker [72], was significantly upregulated on day 3 across all inhibitor treatments, with the strongest effect observed with the combination treatment (Figure 4D). Furthermore, KLF5 mRNA showed non-significant increasing trend, specifically following miR-145-5p inhibition (Figure 4E). Prohibiting (PHB) mRNA also displayed a modest increase on days 3 and 6 under miR-27b-3p inhibition, indicating a supportive role in early differentiation (Figure 4F). PPARγ, a key adipogenic transcription factor [59], was significantly upregulated on days 6, 9, and 12 with the strongest effect observed following the combination treatment (Figure 4G). Expression of LPL, a mature adipocyte marker, increased under both miRNA inhibitor conditions, particularly with miR-27b-3p inhibition (Figure 4H).

Bodipy staining on day 12 confirmed enhanced adipocyte differentiation, revealing increased lipid droplets formation (Figure 4I). The Bodipy-positive area increased from 29% in controls to 34% with miR-145-5p inhibition, 33% with miR-27b-3p inhibition, and 36% with combined treatment (Figure 4J). The number of Bodipy-positive cells also increased significantly, reaching 32% with miR-145-5p inhibition, 31% with miR-27b-3p, and 30% with combined treatment (Figure 4K). These findings demonstrate that inhibition of miR-145-5p and miR-27b-3p enhances adipogenic differentiation, supporting the role of these miRNAs as suppressors of adipocyte maturation.

### miR-145-5p and miR-27b-3p mimics suppress adipogenic differentiation in control cells

**Figure 5:**
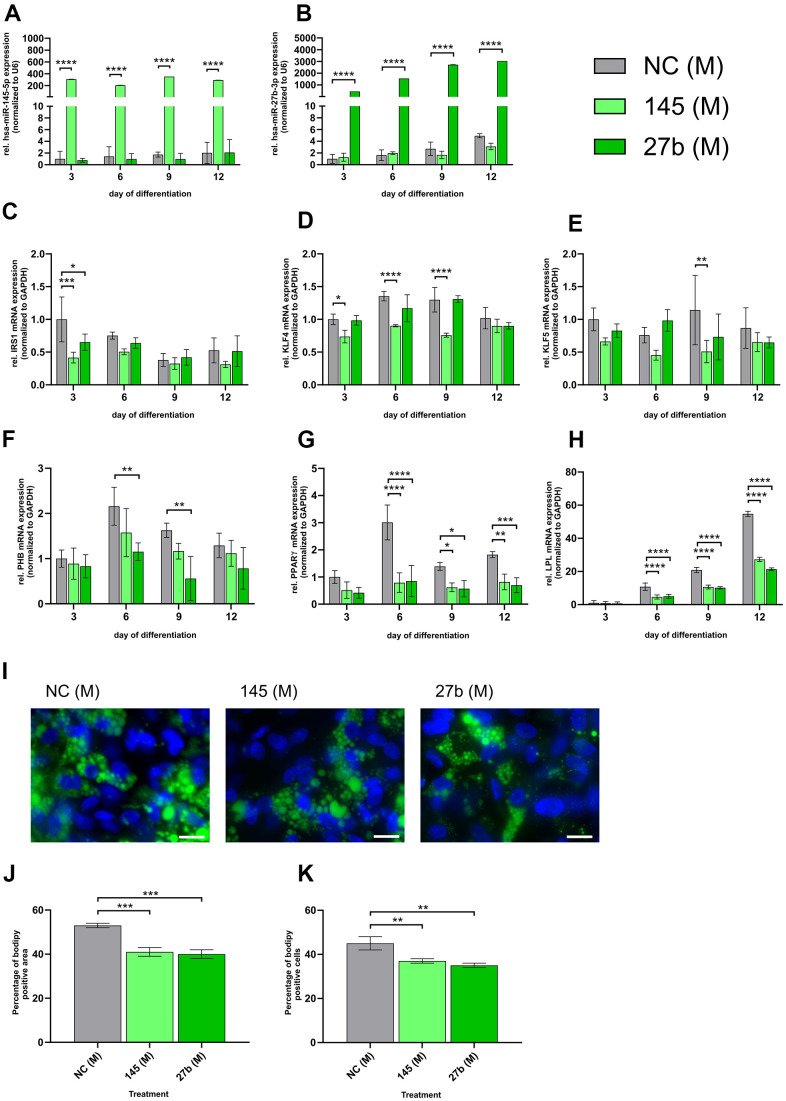
**miR-145-5p and miR-27b-3p mimic suppress adipogenic differentiation in control cells.** Control cell strains (GM05565, GM05757, GM01651) were transfected with 5 nM miR-145-5p or miR-27b-3p mimics on days 0, 3, 6, and 9 during SKP-to-adipocyte differentiation. The treatment groups include 5 nM negative control mimic (NC (M)), 5 nM hsa-miR-145-5p mimic (145 (M)) and 5 nM hsa-miR-27b-3p mimic (27b (M)); qPCR analysis was performed for miR-145-5p (**A**), and miR-27b-3p (**B**) both normalized to U6 as well as for IRS1 (**C**), KLF4 (**D**), KLF5 (**E**), PHB (**F**), PPARγ (**G**) and LPL (**H**) all normalized to GAPDH. (**I**) Bodipy staining (green) of lipid droplets on day 12 of differentiation with representative images (40x magnification; scale bar: 20 μm). DAPI was used as a counterstain. (**J**) Quantification of the percentage of the Bodipy-positive area normalized to the DAPI area. (**K**) Quantification of the percentage of Bodipy-positive cells normalized to the total number of DAPI-positive cells. (**A**–**H**) Values are presented as the mean ± SD (n=3); p > 0.05; * p < 0.05; ** p < 0.01; *** p < 0.001; **** p < 0.0001; two-way ANOVA with Tukey’s multiple comparison test. (**J**, **K**) Values are presented as the mean ± SD (n=3); p > 0.05; * p < 0.05; ** p < 0.01; *** p < 0.001; **** p < 0.0001; Ordinary one-way ANOVA with Dunnett’s multiple comparison test.

To further validate the role of miR-145-5p and miR-27b-3p in impaired adipogenesis observed in HGPS, control SKPs were transfected with 5 nM miR-145-5p and/or miR-27b-3p mimics every 3 days (Figure 5). The effects on miRNA levels and adipogenic markers were assessed through qRT-PCR (Figure 5A–5H).

Both mimics significantly increased miRNA levels, with miR-27b-3p showing a 1000-fold increase compared to 300-fold for miR-145-5p (Figures 5A, 5B). IRS1 levels were significantly reduced as early as day 3, with a more pronounced decrease following miR-145-5p mimic treatment (Figure 5C). KLF4 expression was markedly suppressed on days 3, 6, and 9 by both miRNA mimics, indicating a strong inhibitory effect on early adipogenesis (Figure 5D). KLF5 levels were significantly reduced by the miR-145-5p mimic on day 9, whereas miR-27b-3p showed no substantial effect (Figure 5E). PHB levels were significantly downregulated by the miR-27b-3p mimic on days 6 and 9, whereas the miRNA-145-5p mimic exhibited a milder effect (Figure 5F). PPARγ expression was suppressed by both miRNA mimics on days 6, 9, and 12, demonstrating their inhibitory role in adipogenic differentiation (Figure 5G). Similarly, LPL expression was reduced by approximately 50% following both treatments, with significant downregulation observed on days 6, 9, and 12 (Figure 5H).

Bodipy staining on day 12 confirmed impaired adipogenesis (Figure 5I), with the Bodipy-positive area decreasing from 53% in controls to 41% in miR-145-5p mimic-treated cells and 40% in miR-27b-3p mimic-treated cells (Figure 5J). The percentage of Bodipy-positive cells also declined, decreasing from 45% in control cells to 37% with miR-145-5p and 35% with miR-27b-3p (Figure 5K). Overall, these findings indicate that overexpression of miR-145-5p and miR-27b-3p significantly inhibits adipogenic differentiation by suppressing early and late adipogenic markers and reducing lipid droplet formation, further supporting their role as negative regulators of adipogenesis.

### Proposed model: miR-145 and miR-27b as key regulators of adipogenesis in HGPS

**Figure 6:**
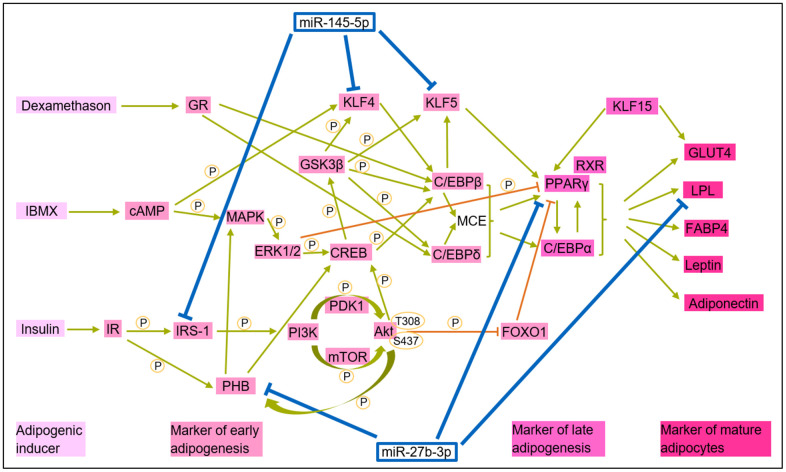
**Integrative model of miR-145-5p and miR-27b in adipogenesis: insights from literature and this study.** Adipogenic proteins are categorized into inducers, early and late markers, and mature adipocyte markers. miR-145-5p and miR-27b-3p impair adipogenesis by targeting key adipogenic markers, disrupting normal differentiation. This graphic used the following studies: [73–80].

Adipocyte differentiation is a highly complex process involving multiple signaling and protein interactions (Figure 6). In HGPS, miR-145-5p and miR-27b-3p are abnormally upregulated, indicating their suppressive role in adipogenesis.

As illustrated in Figure 6 [73–80], miR-145-5p disrupts early and late stages of adipogenesis by suppressing IRS-1 expression, reducing protein kinase B (Akt) phosphorylation [78], and downregulating key adipogenic transcription factors, including C/EBPα and PPARγ [79]. Additionally, miR-145-5p inhibits KLF4 and KLF5. KLF4 not only promotes differentiation but also regulates the cell cycle and apoptosis [74]. In the adipogenic pathway, KLF4 activates C/EBPβ, which in synergy with KLF5, enhances PPARγ activation [75, 76]. Meanwhile, miR-27b-3p targets and inhibits PPARγ, PHB and LPL while indirectly downregulating C/EBPα levels [80]. PHB, a key regulator of adipogenesis, is involved in the phosphoinositide 3-kinase (PI3K)/Akt phosphorylation cascade [77]. The upregulation of miR-145-5p and miR-27b-3p in HGPS disrupts normal adipocyte formation, potentially contributing to the characteristic lipodystrophy observed in patients with HGPS.

## DISCUSSION

The findings comprehensively explored the molecular mechanisms underlying HGPS. Despite advancements in treatments, including the use of lonafarnib, an FTI, certain pathological features persist as symptoms. One such unresolved issue is lipodystrophy, characterized by subcutaneous fat loss [20]. Since adipose tissue is vital for energy storage and thermoregulation, its depletion leads to metabolic disturbances and an increased risk of cardiovascular disease, a leading cause of mortality in HGPS [24–26, 28].

This study utilized global miRNA sequencing to compare miRNA expression profiles between fibroblasts derived from patients with HGPS and control fibroblasts across different stages of the cellular aging process. Our findings reveal distinct miRNA deregulation patterns in HGPS, affecting key cellular processes central to premature aging. To our knowledge, this is the first study to integrate miRNA profiling across both young and aged HGPS and control cell strains, focusing on replicative senescence at various cell passage numbers, reflecting the decline in proliferative capacity [38, 39]. This approach provides critical insights into replicative senescence as a driver of accelerated aging in HGPS. Previously, only one study conducted miRNA expression profiling in HGPS human fibroblasts, where progerin accumulation at increasing passage numbers was presumed to contribute to cellular aging [81].

In the present study, we identified 66 significantly dysregulated miRNAs, including miR-34a-5p, miR-92a-3p, miR-126a, miR-200a, and miR-200b, which regulate target pathways involved in cell cycle control, apoptosis, oxidative stress, and autophagy, all of which are crucial in both normal aging and HGPS pathology [82, 83]. Although, our study also highlights the miR-195/497 and 143/145 clusters, which have been implicated in aging and disease progression [52, 84, 85]. Furthermore, miR-27b-3p and miR-145-5p were found to be upregulated in HGPS fibroblasts, and subsequent target analysis suggests their potential involvement in adipose tissue depletion, a hallmark of HGPS [4, 86, 87].

To investigate the connection between miR-145 and miR-27b dysregulation and impaired adipogenesis, we used a multipotent SKP model, which express lamin A/C, and is highly relevant for HGPS studies [61, 62, 88–91]. Our findings showed a significant reduction in adipocyte differentiation in HGPS derived SKPs compared to control SKPs, indicating severe impairment in the adipogenic process [62, 63]. This study uniquely establishes a link between miRNA regulation and functional adipogenesis, an area that remains underexplored in HGPS research.

Our in-depth analysis showed that miR-145-5p and miR-27b-3p were upregulated in HGPS cells throughout adipogenesis, correlating with the downregulation of key adipogenic transcription factors and markers, including C/EBPα, PPARγ, and FABP4. Notably, similar expression changes in both miRNAs and adipogenic markers were observed in WAT of the Lmna^G609G/G609G^ mouse model [71, 92], a well-established HGPS model. These findings strongly support the association between miRNA dysregulation and lipodystrophy in HGPS [93].

Recent studies further support our findings. Caliskan et al. [94]. identified insulin-like growth factor-binding protein 2 (IGFBP2) as a biomarker in progeria, with miR-27b-3p predicted to target IGFBP2, aligning with our results on miRNA dysregulation and lipodystrophy in HGPS [94]. Moreover, the neural-specific miR-9 has demonstrated protective effects against progerin accumulation, indicating that tissue-specific miRNAs play a role in HGPS pathology [95]. Additionally, miR-29b2/c deficiency has been shown to induce a progeria-like phenotype with adipose tissue reduction, reinforcing the role of miRNAs in adipogenesis and aging [96]. Furthermore, anti-miR-59 treatment in an HGPS mouse model improved adipose tissue maintenance and extended lifespan, supporting the therapeutic potential of miRNA modulation, consistent with our findings on the inhibition of miR-145-5p and miR-27b-3p [97]. Manakanatas et al. showed that miR-34a-5p modulates cellular senescence in progerin-expressing endothelial cells, contributing to cardiovascular pathology via the senescence-associated secretory phenotype (SASP), highlighting the systemic impact of miRNA dysregulation in HGPS [98].

This study provides the first evidence of miR-145-5p and miR-27b-3p as essential regulators of adipogenesis, particularly in HGPS. Inhibition of these miRNAs in HGPS cells enhanced adipogenic differentiation, as indicated by increased expression of IRS1, KLF4, KLF5, PPARγ, and LPL. This was accompanied by an increase in lipid accumulation, as assessed using Bodipy staining. Conversely, miR-145-5p and miR-27b-3p overexpression in control cells suppressed adipogenesis, leading to reduction in key adipogenic markers and lipid accumulation. A limitation of our study is that miRNA deregulation in HGPS is linked to chromatin remodeling changes caused by the aberrant accumulation of progerin hat disrupts nuclear lamina structure and function [33]. Progerin is known to induce chromatin reorganization, including loss of heterochromatin and widespread changes in gene expression. Modulating miR-145 and miR-27b levels may therefore not only affect adipogenic gene networks directly but also influence chromatin accessibility and nuclear lamina integrity, thereby indirectly impacting differentiation [99, 100]. Furthermore, both miRNAs likely regulate additional targets beyond classical adipogenic factors that could contribute to changes in nuclear architecture and epigenetic regulation. These broader effects may play a role in the impaired differentiation phenotype and warrant further investigation.

Overall, this study provides the first comprehensive miRNA profiling of HGPS and control fibroblasts across different stages of cellular senescence. The findings highlight miRNA-145-5p and miRNA-27b-3p as potential therapeutic targets to address adipose tissue defects and premature aging in HGPS. Future research should explore miR-145 and miR-27b’s roles in metabolic disorders such as obesity and diabetes [101–103]. Long-term objectives include the identification of pharmacological compounds capable of modulating these miRNAs, potentially paving the way for novel therapeutic strategies to mitigate metabolic complications associated with HGPS.

## MATERIALS AND METHODS

### Cell lines

Primary human normal dermal fibroblasts were purchased from the Coriell Institute for Medical Research (Camden, NJ, USA). Primary dermal fibroblasts from patients with HGPS carrying the heterozygous LMNA G608G mutation, were purchased from The Progeria Research Foundation Cell and Tissue Bank (http://www.progeriaresearch.org). The following primary human dermal fibroblast cell strains were used in this study: Control cell strains GM01651 (13-year-old female), GM01652 (11-year-old female), GM03349 (10-year-old male), GM05757 (7-year-old male) and GM05565 (3-year-old male), all without mutations; HGPS fibroblast strains: HGADFN003 (2-year-old male), HGADFN127 (3-year-old female), HGADFN164 (4-year old female) and HGADFN178 (6-year-old female, all carrying a heterozygous LMNA c.1824C>T (p.Gly608Gly) mutation in exon 11.

### Mouse model

Transgenic Lmna^G609G^ mice, which phenotypically model HGPS were generously provided by Carlos-Lópes Otin (University of Oviedo, Spain). As previously described [92], all breeding and housing procedures adhered to the Bavarian state regulations and the Animal Welfare Act. This study was approved by the State of Bavaria’s authority (Regierung von Oberbayern, Germany; TVA-ID: 55.2-2532.Vet_01-19-72). The colony was established via embryo transfer under specific pathogen-free (SPF) conditions. To prevent inbreeding, at least five distinct breeding lines were maintained. Mice were housed under controlled conditions (21-22° C, 50% humidity) with species-appropriate enrichment and were provided standard chow (PS RM-H, V1534; ssniff Spezialdiäten GmbH, Soest, Germany). For maintenance breeding, Lmna^+/+^ females were paired with Lmna^G609G/+^ males aged 8 to 20 weeks. Homozygous Lmna^G609G/G609G^ mice were transferred to SPF-grade facilities with equivalent environmental conditions for experimental procedures. To prevent hypothermia, they were provided extra bedding and co-housed with at least one Lmna^+/+^ littermate. Water-soaked chow was provided from 8 weeks of age. For this study, we used female Lmna^+/+^ and homozygous Lmna^G609G/G609G^ mice at the age of 100 -120 days.

### Cell culture

All cells were cultured as monocultures in 10 cm cell culture dishes (Sarstedt, 832472) under standard culture conditions. Cells were grown in high-glucose Dulbecco’s Modified Eagle Medium (DMEM; Thermo Fisher-Gibco, Waltham, MA, USA, D6429, containing 4.5 g/L glucose), supplemented with 15% fetal bovine serum (FBS; Thermo Fisher-Gibco, 10270106), 1% L-glutamine (Thermo Fisher-Gibco, 25030081), 1% penicillin-streptomycin (Thermo Fisher-Gibco, 1514022), and 0.5% gentamicin (Thermo Fisher-Gibco, 15710049) in a cell incubator (Binder, Tuttlingen, Germany, 9140-0046) at 37° C and 5% CO_2_. For sub-culturing, cells were first washed with phosphate-buffered saline (PBS; Sigma-Aldrich, D8537), trypsinized using 2 mL trypsin-EDTA (trypsin-ethylenediaminetetraacetic acid, Thermo Fisher – Gibco, 25200-056), and distributed into new dishes after stopping the reaction. The culture medium was changed every 2-3 days. Monocultures with a degree of senescence level of < 5% were classified as young, whereas those with > 15% senescence were considered old. For SKP isolation, the cells were used at 80% confluence and a senescence of 5%. The levels of replicative senescence used in experiments are detailed in Supplementary Table 3.

### Senescence associated β-galactosidase assay

Senescence was evaluated in control and HGPS fibroblast cultures at each subculture using the senescence-associated β-galactosidase assay (SA-β-Gal) as previously described by Dimri et al*.* (1995) [104]. Cells were first washed with PBS for 5 min and then fixed at room temperature for 5 min using a fixation solution containing 0.2% glutaraldehyde (Sigma-Aldrich, St. Louis, MO, USA, G5882) and 2% formaldehyde (Sigma-Aldrich, 104003). After fixation, cells were washed twice with PBS for 5 min and subsequently incubated overnight at 37° C in absence of CO_2_ with an SA-β-Gal staining solution. This solution contained 5 mM potassium ferricyanide (III) (Merck KGaA, 104973, Darmstadt, Germany), 5 mM potassium ferrocyanide (II) (Sigma-Aldrich, P9387), 2 mM magnesium chloride (MgCl_2_, Sigma-Aldrich, M-1028), 150 mM sodium chloride (NaCl, Sigma-Aldrich, 14 310166), 0.5 mg/mL 5-bromo-4-chloro-3-indolyl-β-D-galactopyranoside (X-gal, Sigma-Aldrich, 3117073001), and 40 mM citrate/sodium phosphate buffer (pH 6.0). At least 1000 cells were counted per sample, and cells exhibiting blue staining were classified as senescent.

### Genotyping

Genetic material was extracted from earmark punches collected at weaning using a homemade DNA extraction kit (10XStandard reaction buffer with MgCl_2_ [Biotools B&M Labs, SA, P0098], DNA Polymerase [Biotools B&M Labs, SA, P0116], dNTP MIX [Biotools B&M Labs, SA, P0066]) [92]. The extraction was performed using the Mixing Block MB-102 (BIOER, Hangzhou, China) at 95° C. PCR was performed using previously published primers: DNA-Mm-*Lmna* forward, 5′-GGTTCCCACTGCAGCGGCTC-3′, and DNA-Mm-*Lmna* reverse, 5′-GGACCCCACTCCC-TTGGGCT-3 [71]. Amplification followed a previously described protocol [105] using the ICycler PCR System (Bio-Rad iCycler iQ™), with initial denaturation at 94° C for 10 min followed by 35 cycles at 94° C for 1 min, 64° C for 1 min, and 72° C for 1 min, with a final extension at 72° C for 7 min.

### White adipose tissue (WAT) harvesting

Experimental Lmna^G609G/G609G^ mice and age-matched Lmna^+/+^ controls were euthanized by cervical dislocation under 5% isoflurane anesthesia and perfused with 20 mL PBS (Sigma Aldrich, St. Louis, MO, USA). WAT was collected for RNA isolation, immediately frozen in liquid nitrogen, and stored at -80° C until further processing.

### RNA extraction and purification

Cells were washed with PBS, trypsinized with trypsin-EDTA, and collected after stopping the reaction with a medium containing 15% FBS. Collected cells were centrifuged at room temperature for 5 min at 1200 rpm, and the cell pellets were frozen in liquid nitrogen after removing the supernatant. Total RNA, including microRNAs from pelleted cells or mouse WAT tissue, was isolated using the miRNeasy Mini Kit protocol (Qiagen, 217004). Briefly, collected cell pellets or mouse WAT were lysed and homogenized using 700 μl QIAzol lysis reagent, and the homogenate was incubated for 5 min at room temperature (RT). Thereafter, 140 μl Chloroform was added, mixed well, and incubated at RT for 3 min before centrifuging at 12000x g for 15 min at 4° C. The upper aqueous phase was transferred to a new collection tube, and 1.5 volumes of 100% ethanol were added. The solution was mixed thoroughly, transferred into a RNeasy® Mini column with a collection tube, and centrifuged at 8000x g for 1 min at 4° C. The column was washed once with 700 μl RWT buffer and twice with 500 μl RPE buffer. Each washing step included centrifugation at 8000x g for 1 min at 4° C. After a final washing step, the column was centrifuged at 8000x g for 2 min at 4° C, and completely dried by transferring it to a new collection tube and centrifuging at 13000x g for 1 min at 4° C. RNA was eluted by adding 30 μL RNase-free water and centrifuged at 8000x g for 1 min at 4° C. RNA concentration measurements were performed using 1 mM Tires buffer, pH 7,0 (T1503, Sigma), whereas quality control was performed using 1 mM Tris buffer, pH 7.5 (T1503, Sigma). The quantity and quality of isolated RNA were measured by using a NanoDrop spectrophotometer (NanoDrop ND-1000 V3. 8.1, Thermo Fisher). RNA samples with 260/280nm absorbance ratio between 1.9 and 2.1 were considered to be of good purity.

### Sequencing

For global miRNA sequencing, both young and old passage cells were selected from each of the following cell strains. Young passage (Supplementary Table 3) included P14 to P18 for control and P11 to P15 for HGPS with relative senescence <5%. Old passages (Supplementary Table 3) included P22 to P26 for control and P15 to P23 for HGPS with senescence between 15 and 20%. The control cell strains included GM01651 (13-year-old female), GM01652 (11-year-old female) and GM03349 (10-year-old male), all without mutations, and HGPS cell strains included HGADFN003 (2-year-old male), HGADFN127 (3-year-old female) and HGADFN178 (6-year-old female) all carrying heterozygous c.1824C >T (p.Gly608Gly) mutation in *LMNA* Exon 11. Total RNA, including miRNAs, was isolated from these cells and assessed for total RNA concentration and purity as described above. RNA integrity was evaluated by loading at least 200 ng of RNA onto a denaturing agarose gel. Total RNA was extracted using a Trizol reagent (Invitrogen, Carlsbad, CA, USA), and its quality and quantity were analyzed using a Bioanalyzer 2100 (Agilent, Santa Clara, CA, USA) ensuring an RNA integrity number >7.0. Approximately 1 ug of total RNA was used to prepare a small RNA library following to the TruSeq Small RNA Sample Prep Kits protocol (Illumina, San Diego, USA). Single-end sequencing of 50 bp on an Illumina HiSeq 2500 at the LC Sciences (Hangzhou, China). miRNA-seq data have been deposited at the NCBI GEO as under accession number GSE282307 and are publicly available as of the date of publication.

### Processing of sequencing data

Raw sequencing reads were analyzed using ACGT101-miR (LC Sciences, Houston, TX, USA) to remove adapter dimers, junk sequences, low-complexity reads, common RNA families (rRNA, tRNA, snRNA, snoRNA), and repeat sequences. Unique sequences ranging from 18 to 26 nucleotides in length were mapped to human precursors in miRBase 22.0 (http://mirbase.org/) via BLAST search to identify both known miRNAs and novel 3p- and 5p-derived miRNAs. Alignment allowed for length variation at both the 3’ and 5’ ends, as well as a single mismatch within the sequence. Unique sequences that mapped to mature miRNAs-containing arms of known human precursor hairpins were classified as known miRNAs (http://mirbase.org/). Sequences mapping to the opposite arm of a known human precursor hairpin, related to the annotated mature miRNA were considered novel 5p- or 3p-derived miRNA candidates. The mapped pre-miRNAs were further analyzed through BLASTed (http://rna.tbi.univie.ac.at/cgi-bin/RNAWebSuite/RNAfold.cgi) to determine their genomic locations. Normalization of sequence counts was performed following the method described by Dillies et al. [106]. All expressed miRNAs were listed, and sequence counts in each sample were normalized by dividing the counts by the library size parameter of the corresponding sample. The library size parameter was calculated as the median value ratio between the counts of a specific sample and a pseudo-reference sample, where the pseudo-reference sample was the geometric mean across all samples. To test for differential miRNA expression between young and old control and HGPS primary dermal fibroblasts, the 1019 identified miRNAs (915 known and 104 novel miRNAs) were further analyzed using a DESeq2 software within the R statistical environment (R version 4.1.1 (2021-08-10 R Foundation for Statistical Computing, Vienna, Austria; URL https://www.R-project.org)) for normalization and filtering. All miRNAs were analyzed for all possible comparisons between all conditions while accounting for batch effects.

The lists of differentially expressed miRNAs generated by DESeq2 were further filtered to remove miRNAs with low read counts (fewer than 10 reads in at least two of the three biological replicates in both conditions being compared), as these were considered of low biological relevance. The general cut-off criteria of differentially expressed miRNAs were a p-value of 0.05 and a q-value of 0.1, as described previously by Osabe et al. (2021) [107].

### Skin-derived precursor (SKP) isolation

SKPs were isolated from human primary dermal fibroblasts when culture reached 80% confluence and senescence levels were < 5%. The isolation method followed the low pH stress protocol established by Budel and Djabali in 2017, [61] using acidic Hank’s Balanced Salt Solution (HBSS; Thermo Fisher-Gibco, 14175053) adjusted to pH 5.7 with hydrochloric acid (HCL; Merck KGaA, Darmstadt, Germany, 1.00319.2500). Cells were first washed with PBS and trypsinized using trypsin-EDTA. Detached cells were collected, pelleted via centrifugation at 1200 rpm for 5 min at RT, and washed with PBS. Cell counts and viability assessments were performed using a Muse Cell Analyzer (Merck Millipore, Burlington, MA, USA). Every one million cells were resuspended in 500 μL HBSS (pH 5.7), and incubated for 25 min at 37° C with resuspension every 5 min. Thereafter, cells were centrifuged at 1200 rpm for 5 min at RT, resuspended with 6 mL SKP medium, and evenly separated into two T25 non-tissue-culture-treated flasks (Fisher Scientific-Falcon, Hampton, NH, USA, 10112732) as previously described [61]. The SKP culture medium consisted of a 3:1 mixture of DMEM low glucose (1g/L glucose; Thermo Fisher-Gibco, 21885025) and F12 (DMEM/F12, Thermo Fisher-Gibco, 21765029), supplemented with 2% B27 (Thermo Fisher—Gibco, 17504044), 1% penicillin-streptomycin (Thermo Fisher-Gibco, 1514022), 0.2% fungizone (Thermo Fisher-Gibco, 15290018), 0.02% epidermal growth factor (EGF; Thermo Fisher-Gibco, PHG0311), and 0.04% basic fibroblast growth factor (bFGF; Thermo Fisher-Gibco, PHG0026). SKPs were cultured for 5 days in SKP medium, with daily resuspension to prevent spheroid adhesion. On day 2 and 4, cells were fed with 10x SKP medium, which contained ten times the concentration of B27, EGF, and bFGF, diluted to a final concentration of 1x in SKP culture medium.

### Adipocyte differentiation

SKPs were collected on day 5 and centrifuged at 1200 rpm for 5 min at RT. The resulting SKP spheroids were washed twice with PBS (Sigma-Aldrich, D8537) and dissociated using 4 ml trypsin (trypsin-ethylenediaminetetraacetic acid, Thermo Fisher-Gibco, 25200-056). Cell count and viability were assessed using a Muse Cell Analyzer (Merck Millipore, Burlington, MA, USA). The dissociated cells were then seeded into 24-well plates (Sarstedt, 833922) containing coverslips (VWR, 43233819) at a density of 1.2 x 10^5^ cells/well. Cells were cultured following protocols from Budel and Djabali [61] and Najdi et al*.* [62], using adipocyte differentiation medium. The medium consisted of three volumes of high glucose DMEM (4.5 g/L glucose, Thermo Fisher-Gibco, 21885025) and one volume of F12 (DMEM/F12 in a 3:1 ratio; Thermo Fisher-Gibco, 21765029). This was supplemented with 10% FBS (Thermo Fisher-Gibco, 10270106), 1% penicillin-streptomycin (Thermo Fisher-Gibco, 1514022), 1% insulin (Sigma-Aldrich, I2643, stock in 0.01M HCL [Merck KGaA, 1.00319.2500]), 1% 3-isobutyl-1-methylxanthine (IBMX, Sigma-Aldrich, St., Louis, MO, USA, I7018, stock in absolute ethanol [VWR Chemicals, Radnor, PA, USA, 20821.33]), 1% L-ascorbic acid (Sigma-Aldrich, A8960, stock in Ultra-Pure water from MilliQ), 0.4% dexamethasone (Dexa, Sigma-Aldrich, D4902, stock in absolute ethanol), 0.2% fungizone (Thermo Fisher-Gibco, 15290018), and 0.125% indomethacin (Sigma-Aldrich, I7378, stock in 100% DMSO (Sigma-Aldrich, D2650). The differentiation process continued for 12 days, with medium changed every three days.

### Transfection assays

Transfection assays were performed immediately after seeding SKPs into 24-well plates. The cells were transfected using the Interferin® (Polyplus, 101000028) transfection reagent, following the manufacturer’s protocol. For the miRNA mimic experiment, hsa-miR-145-5p and hsa-miR-27b-3p (mirVana® miRNA mimic, Thermo Fisher, 4464066) were transfected into control cell strains (GM05565, GM05757, GM01651) at a concentration of 5 nM. Conversely, HGPS cells (HGADFN003, HGADFN127, HGADFN164) were transfected with 10 nM miRNA inhibitors for hsa-miR-145-5p and hsa-miR-27b-3p (mirVana® miRNA inhibitor, Thermo Fisher, 4464084). As controls, cells were transfected with 5 nM of a negative mimic control (mirVana™ miRNA Mimic, Negative Control #1, Thermo Fisher, 4464058) or 10 nM of a negative inhibitor control (mirVana™ miRNA Inhibitor, Negative Control #1, Thermo Fisher, 4464076). The transfection mix for each well was prepared by combining either a 5 nM miRNA mimic or 10 nM miRNA inhibitor with 1 μL of Interferin® (Polyplus, 101000028) in high-glucose DMEM e (4.5 g/L glucose; Thermo Fisher-Gibco, 21885025) supplemented with 1% L-glutamine (Thermo Fisher-Gibco, 25030081), 1% penicillin-streptomycin (Thermo Fisher-Gibco, 1514022), and 0.5% gentamicin (Thermo Fisher-Gibco, 15710049). After 10 min of incubation,100 μL of the transfection mix was added to each well and left to interact with the cells for 3 days. Transfections were repeated every three days to maintain gene silencing efficacy.

### Reverse transcription (RT) and quantitative polymerase chain reaction (qPCR)

A total of 500 ng RNA was reverse transcribed into cDNA using a High-Capacity cDNA Reverse Transcription (RT) Kit (Thermo Fisher, 4368814) with 10x RT buffer, 25x dNTP, and 10% reverse transcriptase. For total cDNA synthesis, 10x random primers from the kit were used. For miRNA, transcription, RT stem-loop primers with eight complementary nucleotides were designed using snRNAprimerDB [108]. All primers were purchased from Thermo Fisher (10336022) and are listed in Supplementary Table 4. For the ICycler PCR System (Bio-Rad iCycler iQ™), stem-loop reverse transcription of miRNAs was performed under the following thermal cycling conditions: initial incubation at 16° C for 30 min followed by 60 cycles of 30 s at 30° C, 30 s at 42° C and 1 min at 50° C. The reaction was then held at 85° C for 5 min and terminated at 4° C. For random reverse transcription, the reaction was set at 25° C for 10 min, followed by 37° C for 120 min, 85° C for 5 min and termination at 4° C.

Quantitative real-time PCR (qPCR) was performed using PowerUP SYBR Green Master Mix (Applied Biosystems™, Thermo Fisher). The thermal cycling conditions for miRNA amplification involved uracil-DNA glycosylase (UGD) activation at 50° C for 2 min, followed by Dual-Lock DNA polymerase activation at 95° C for 2 min. This was followed by 60 cycles of denaturation at 95° C for 15 s and annealing at 60° C for 1 min. The qPCR for total RNA included an initial activation step at 50° C for 2 min, pre-soak at 95° C for 10 min and 40 cycles of denaturation at 95° C for 15 s and annealing at 60° C for 1 min.

miRNA levels were normalized to U6 small nuclear RNA, whereas glyceraldehyde-3-phosphate dehydrogenase (GAPDH) was used for normalizing of target mRNA expression. Primer pairs for all evaluated genes were purchased from Thermo Fisher (Supplementary Table 4). All experiments were performed in triplicates.

### Western blotting

For western blotting, cells were collected by scraping the culture plates. After washing with PBS (Sigma-Aldrich, D8537), cells were lysed in a 1:1 mixture of Laemmli buffer and lysis buffer. The Laemmli buffer consisted of 4x Laemmli sample buffer (Bio-Rad Laboratories, 1610747), 6% 2-Mercaptoethanol (Bio-Rad Laboratories, 161-0710), 1% protease-inhibitor (Thermo Fisher, WF326480), 1% phosphatase-inhibitor (Thermo Fisher, TG269618), and 0.75% phenylmethylsulfonyl fluoride (200 mM PMSF, Sigma-Aldrich, 10837091001). The lysis buffer contained 150 mM NaCl (Sigma-Aldrich, 310166), 1% Triton X-100 (Sigma-Aldrich, T9284), 50 mM Tris (Sigma Aldrich, SLBR620IV), 1% sodium dodecyl sulfate (SDS, Sigma-Aldrich, L3771-100G), and 1 mM EDTA (Sigma-Aldrich, E9884), adjusted to pH 8.0. Proteins were separated via SDS-PAGE in a 15% acrylamide gel (Bio-Rad Laboratories, 161057) and transferred via wet transfer onto nitrocellulose membranes (Amersham Protran Premium Western blotting membrane, Sigma-Aldrich, GE10600003) using a wet transfer system. To normalize for total protein content, trichloroethanol (Sigma-Aldrich, T54801) was added, and gels were imaged immediately after electrophoresis.

The efficiency of protein transfer was assessed using reversible Ponceau S staining (Sigma-Aldrich, P7170). Membranes were then blocked with 5 % non-fat milk (Sigma-Aldrich, P7170, T145.3) for 1 h, at RT, followed by overnight incubation with primary antibodies at 4° C. After three washes with Tris-buffered saline with 0.1% Tween 20 (Sigma-Aldrich, P9416) for 5 min each, membranes were incubated with horseradish peroxidase (HRP)-conjugated secondary antibodies for 1 h, at RT. Protein detection was performed using Clarity^TM^ Western ECL substrate (Bio-Rad, 1705061), and chemiluminescence signals were captured using ChemiDoc^TM^ MP Imaging System (Bio-Rad Laboratories). Densitometric analysis was conducted with Image Lab Software (version 6.1.0, Bio-Rad Laboratories). Protein levels were normalized to that of GAPDH.

### Bodipy staining

Cells grown on 12 mm glass coverslips (VWR, 43233819) were washed once with PBS and fixed with 2 % paraformaldehyde (PFA, Sigma-Aldrich, P6148) for 10 min at RT. Then, cells were washed with PBS and incubated with Bodipy (Invitrogen, Waltham, MA, USA, D3922, 1:5000 dilution) for 45 min at RT to stain lipid droplets. Following incubation, coverslips were washed with PBS for 8 min and counterstained with DAPI Vectashield mounting medium (Vector Laboratories, H-1200). Images were acquired using an Axio Imager D2 fluorescence microscope (Light source: X-cite 120Q (EXFO Photonic Solutions Inc., Mississauga, ON, Canada); objectives used: EC-Plan Neofluar 10×/0.3 (420340-9901, Carl Zeiss), Plan-Apochromat 40×/0.95 Korr (440654-9902, Carl Zeiss); camera used: AxioCam MRm (Carl Zeiss, Oberkochen, Germany); excitation and emission filters used: filter set 49 (424931, Zeiss), filter set 38 HE (424931, Zeiss)). Images were captured using AxioVs40 V 4.8.2.0 software (Carl Zeiss, Oberkochen, Germany).

### Image analysis

Fiji software (ImageJ, version 2.14.0/1.54f, Java 1.8.0_322, Wayne Rasband, NIH) was used for image analysis [109]. During image processing, only the brightness and contrast were adjusted to quantify the number of Bodipy-positive cells and Bodipy intensity, while all other parameters remained unchanged. For Bodipy intensity quantification, images acquired at 10x magnification were analyzed using the RenyiEntropy auto threshold method. The threshold was applied independently to both Bodipy and DAPI signals. For Bodipy-positive cell counting, images captured at 40x magnification were used. A minimum of 1000 cells were counted, with cells surrounded by lipid droplets classified as Bodipy positive. For the quantification, Bodipy signal intensity was normalized to the DAPI signal, while the number of Bodipy-positive cells was normalized to the total number of cells per image. Figure illustrations were created using Inkscape (Version 1.1.1 (3bf5ae0d25, 2021-09-20), GPL).

### Quantification and statistical analyses

For statistical analyses, the following tests were performed using GraphPad Prism (Version 8.0.2 (263), San Diego, CA, USA). An unpaired *t*-test was used to compare the percentage of BODIPY-positive area/cells in HGPS cells versus control cells, as well as gene expression levels in LMNA^(G609G/G609G)^ mice compared to LMNA^(+/+)^ mice. A two-way ANOVA with Sidak's multiple comparisons test was applied to examine gene expression across different differentiation days between HGPS and control cell strains. To evaluate the effects of different treatments on gene expression, a two-way ANOVA with Tukey's multiple comparisons test was performed, comparing each treatment to the negative control group at the same time point. For the quantification of Body-positive areas and cell counts under different treatments, a one-way ANOVA with Dunnett's multiple comparison test was conducted, comparing each treatment group to the control group. For senescence analysis and BODIPY-positive cell counting, a minimum of 1000 cells per strain and condition were analyzed. Results are presented as the mean ± standard deviation (SD). Statistical significance was indicated as follows: p > 0.05 (not significant); * p < 0.05; ** p < 0.01; *** p < 0.001; **** p < 0.0001.

## Supplementary Material

Supplementary Figures

Supplementary Table 1

Supplementary Tables 2-4
