# Supplementary material for: Deregulated miR-145 and miR-27b in Hutchinson-Gilford progeria syndrome: implications for adipogenesis
Source: Aging (Albany NY). 2025 Aug 27;17(9):2278–311. doi: 10.18632/aging.206309 (PMC12517220; doi:10.18632/aging.206309)
Supplement: Supplementary Figures [file aging-17-9-206309-s001.pdf]

SUPPLEMENTARY FIGURES

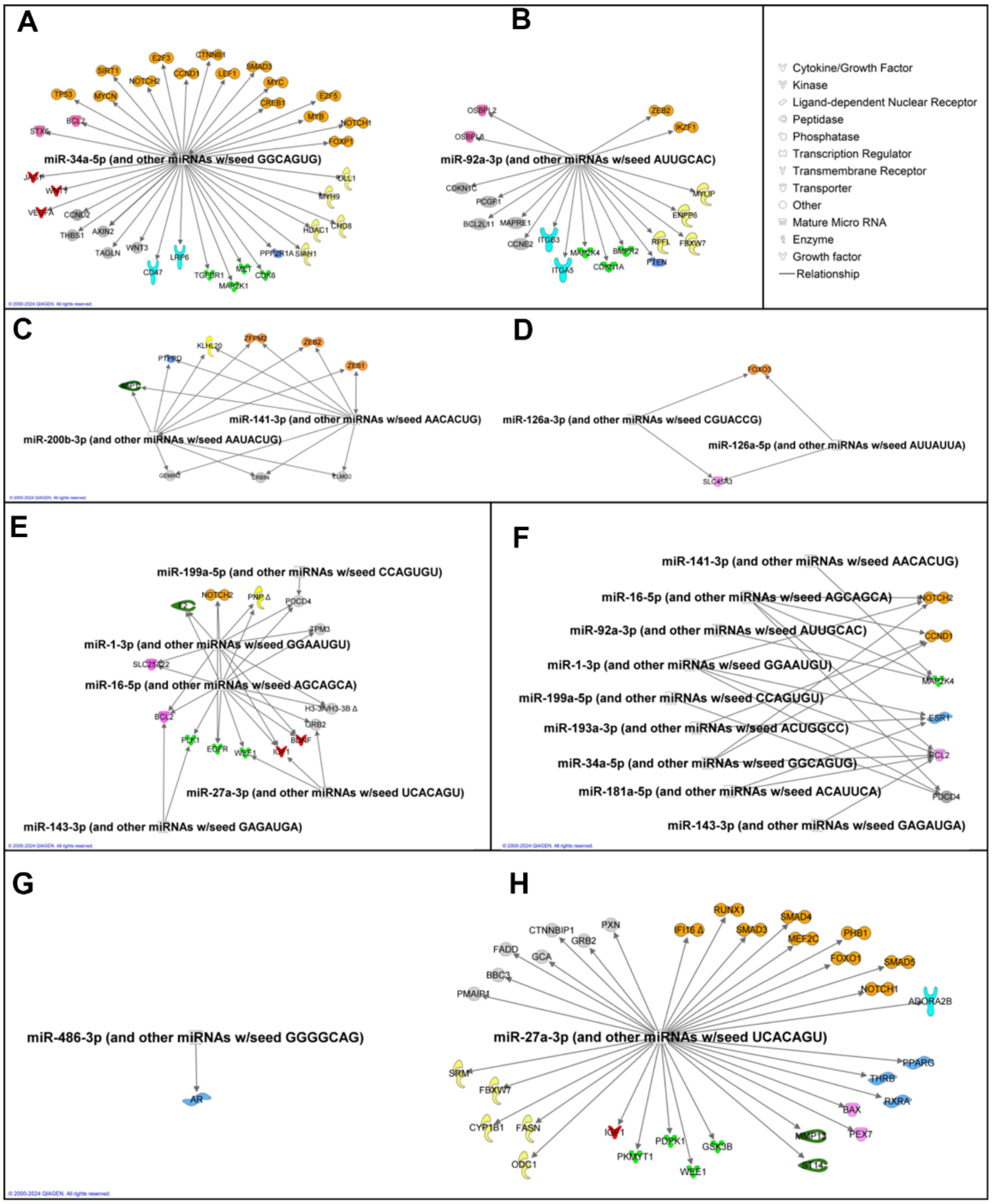

Supplementary Figure 1. Connection of deregulated miRNAs to (premature) aging. Link of miRNAs to normal aging (**A**, **B**). Role of miR-34a-5p (**A**) and miR-92a-3p (**B**) in cellular aging. Differentially expressed miRNAs and their experimentally observed targets (IPA) by comparing old control cells (15-20% senescence) to young control cells (senescence <5%). Link of miRNAs to HGPS cellular aging (**C**, **D**). miR-200a/b (**C**) and miR-126a-3p/5p (**D**) set of differentially expressed miRNAs and their experimentally observed, overlapping targets (IPA) by comparing old HGPS cells (15-20% senescence) to young HGPS cells (senescence <5%). Link of miRNAs to early changes in HGPS (**E**). Differentially expressed miRNAs and their experimentally observed overlapping targets (IPA) by comparing young HGPS cells (Senescence <5%) to young control cells (Senescence <5%). Link of miRNAs to premature aging (**F**). Differentially expressed miRNAs and their experimentally observed overlapping targets (IPA) by comparing old HGPS cells (15-20% senescence) to old control cells (15-20% senescence). miRNAs driving early changes in HGPS young cells (**G**, **H**). Differentially expressed miRNA miR-486-3p (**G**) and miR-27b-3p (**H**) with their experimentally observed targets (IPA). miR-200a-3p illustrated as miR-141-3p (and other miRNAs w/seed AACACUG); miR-195-5p and miR-497-5p are combined, and both are shown as miR-16-5p (and other miRNAs w/seed AGCAGCA); miR-199b-5p illustrated as miR-199a-5p (and other miRNAs w/seed CCAGUGU); miR-27b-3p illustrated as miR-27a-3p (and other miRNAs w/seed UCACAGU).

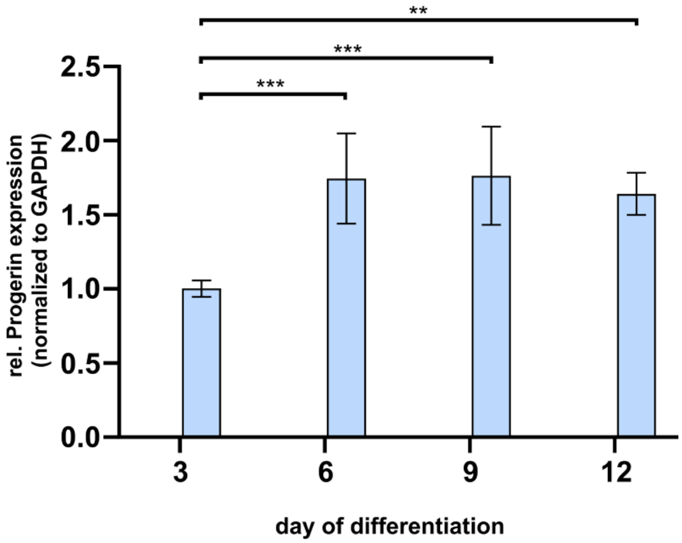

Supplementary Figure 2. Quantification of progerin at different differentiation stages. Representative western blot of Lamin A/C shown in Figure 2E. Distinct progerin band exclusively in HGPS samples. Quantification of progerin protein level of HGPS samples normalized to GAPDH on day 3, 6, 9, and 12 of differentiation. Values are presented as the mean ± SD (n=3); p > 0.05; * p < 0.05; ** p < 0.01; *** p < 0.001; **** p < 0.0001; two-way ANOVA with Tukey's multiple comparisons test.

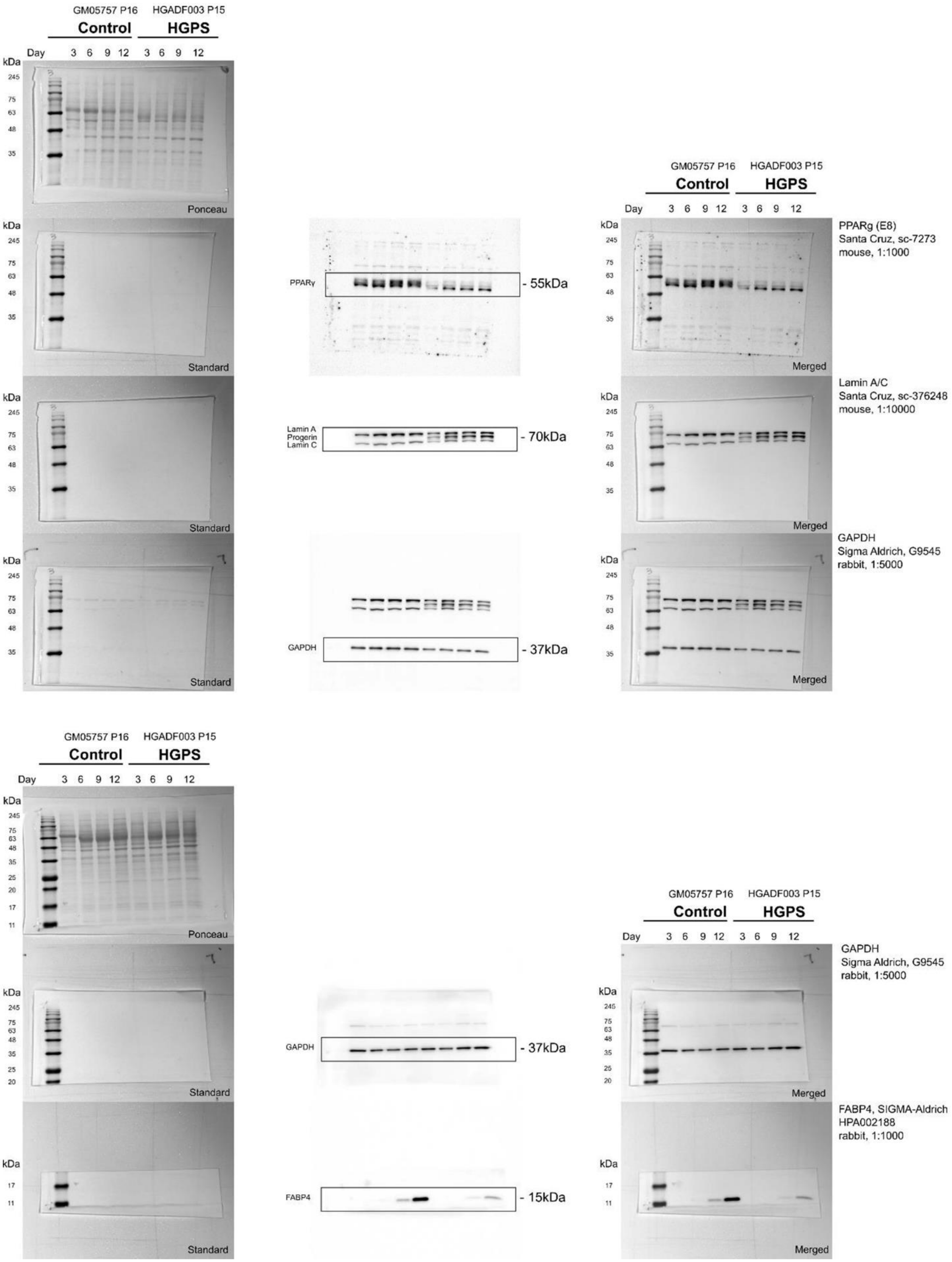

Supplementary Figure 3. Fully uncropped and unprocessed images. Lanes of the blots are labeled as they are in Figure 2E, the place where cropping was applied is marked (with a box).
