# Supplementary material for: Deregulated miR-145 and miR-27b in Hutchinson-Gilford progeria syndrome: implications for adipogenesis
Source: Aging (Albany NY). 2025 Aug 27;17(9):2278–311. doi: 10.18632/aging.206309 (PMC12517220; doi:10.18632/aging.206309)
Supplement: Supplementary Table 1 [file aging-17-9-206309-s002.docx]

**Supplementary Table 1. Overview of miRNA sequencing data.**

Differentially expressed miRNAs across all six possible comparisons between the different conditions (control old vs. control young; HGPS old vs. HGPS young; young HGPS vs. young control; old HGPS vs. old control; old HGPS vs. young control; young HGPS vs. old control) including log2FoldChange, pvalue, and qvalue. Genome-wide miRNA profiles of HGPS and control fibroblast cultures of a young passage with relative senescence < 5 % and an old passage of senescence between 15 and 20 %; control cell strains: GM01651c, GM01652c, GM03349c; HGPS cell strains: HGADFN003, HGADFN127, HGADFN178.
