# Supplementary material for: Deregulated miR-145 and miR-27b in Hutchinson-Gilford progeria syndrome: implications for adipogenesis
Source: Aging (Albany NY). 2025 Aug 27;17(9):2278–311. doi: 10.18632/aging.206309 (PMC12517220; doi:10.18632/aging.206309)
Supplement: Supplementary Tables 2-4 [file aging-17-9-206309-s003.pdf]

Supplementary Table 2. Link of experimentally observed targets for differentially expressed miRNAs to canonical pathways and cellular functions.

Targets	Association	Reference
**link of miRNAs to normal aging – miR-34a-5p**		
Axin2, Wnt family members (WNT1, WNT3), β-catenin (CTNNB1)	Wnt signaling pathway: cell renewal and tissue homeostasis	[1, 2]
Jagged1 (JAG1), Notch receptors NOTCH1, NOTCH2), delta-like ligands (DLL1)	Notch signaling pathway: cell differentiation and senescence	[3]
p53, sirtuin 1 (SIRT1)	modulators of the aging processes: regulate DNA damage responses, apoptosis, and survival	[4]
mitogen-activated protein kinase kinase 1 (MEK1), SMAD3, and TGF beta receptor 1 (TGFBR1)	aging-associated pathways: MAPK/ERK and TGF-β pathways: cell proliferation and repair	[5]
MYC, MYCN	Proto-oncogenes: cellular metabolism and growth	[6]
Histone deacetylase 1 (HDAC1) and chromodomain helicase DNA binding protein 8 (CHD8)	epigenetic regulators: gene expression: chromatin state, DNA methylation, histone modification, chromatin remodeling	[7, 8]
**link of miRNAs to normal aging – miR-92a-3p**		
cyclin E2, cyclin-dependent kinase inhibitors 1A and 1C, F-box and WD repeat domain-containing 7 (FBXW7)	cell cycle regulation, maintenance of cellular homeostasis: stem cell aging	[9, 10]
BCL2-like 11 (BCL2L11)	apoptosis, cell proliferation	[11]
phosphatase and tensin homolog (PTEN)	modulator of survival	[12]
Polycomb group proteins (PCGF1, ZEB2)	chromatin remodeling	[13, 14]
Integrins (ITGA5, ITGB3) and bone morphogenetic protein receptor type 2 (BMPR2)	inflammation and age-related fibrosis	[15–17]
oxysterol binding proteins (OSBPL2, OSBPL8)	lipid metabolism; oxidative stress	[18]
mitogen-activated protein kinase kinase 4 (MAP2K4)	MAPK pathway: stress response and senescence	[19]
**link of miRNAs to HGPS cellular aging – miR-126-3p; miR-126-5p**		
forkhead box O3 (FOXO3)	key longevity factor: oxidative stress responses, autophagy, apoptosis: helping protect cells from age-related damage	[20, 21]
solute carrier family 45 member 3 (SLC45A3)	immune cell infiltration, cellular homeostasis	[22]
**link of miRNAs to HGPS cellular aging – miR-200a/b**		
BRCA1-associated protein 1 (BAP1)	chromatin remodeling, DNA repair, and genomic stability	[23, 24]
ERBB2 interacting protein (ERBIN), engulfment and cell motility 2 (ELMO2)	cell growth, polarity, motility, and cytoskeletal organization, tissue regeneration	[25–28]
GEM nuclear organelle-associated protein 2 (GEMIN2)	nuclear structure	[29, 30]
zinc finger E-box binding homeobox 1 and 2 (ZEB1, ZEB2)	stem cell differentiation, epithelial-to-mesenchymal transition (EMT): tissue repair and fibrosis	[31, 32]
Kelch like family member 20 (KLHL20)	protein homeostasis: counteracting age-related cellular damage	[33, 34]
**link of miRNAs to early changes in HGPS**		
B-cell lymphoma 2 (BCL2), programmed cell death 4 (PDCD4)	apoptosis modulators: cell death and tissue degeneration	[35–37]
brain-derived neurotrophic factor (BDNF), epidermal growth factor receptor (EGFR), insulin-like growth factor 1 (IGF-1), growth factor receptor bound protein 2 (GRB2)	cellular growth and repair: tissue regeneration and neurodegeneration with age	[38–42]

H3.3 histone A	chromatin structure and gene expression: cellular senescence	[43–46]
Polo-like kinase 1 (PLK1), WEE1 G2 checkpoint kinase (WEE1)	cell cycle, maintaining genomic stability	[47, 48]
**link of miRNAs to premature aging**		
BCL2, PDCD4	apoptosis regulators: cell death and tissue degeneration	[35–37, 49]
Cyclin D1 (CCND1)	cell cycle, senescence	[50, 51]
MAP2K4	RAS/MAPK signaling pathway: cell proliferation and survival, senescence	[52]
NOTCH2	cell adhesion, differentiation, and tissue homeostasis	[53, 54]

Target analysis of genome-wide miRNA sequencing of HGPS and control fibroblast cultures of a young passage with relative senescence < 5 % and an old passage of senescence between 15 and 20 %; control cell strains: GM01651c, GM01652c, GM03349c; HGPS cell strains: HGADFN003, HGADFN127, HGADFN178.

Supplementary Table 3. Passage numbers of young and old cell cultures for each primary fibroblast cell strain.

Cell Strain	Young culture senescence < 5%	Old culture senescence 15-20%
GM01651	Passage 14-17	Passage 23-25
GM01652	Passage 15-18	Passage 24-26
GM03349	Passage 16-18	Passage 22-26
GM05757	Passage 15-17	-
GM05565	Passage 14-18	-
HGADFN003	Passage 12-15	Passage 21-23
HGADFN127	Passage 11-14	Passage 18-19
HGADFN178	Passage 11-13	Passage 15-17
HGADFN164	Passage 10-14	-

Supplementary Table 4. List of primer pairs with forward (F), reverse (R), and reverse transcription (RT) primer sequences.

Target gene	Primer sequence (5' to 3')
**mmu-miR-145a-5p**	RT: GTCGTATCCAGTGCAGGGTCCGAGGTATTCGCACTGGATACGACAGGGATTC<br>F: AACAAGGTCCAGTTTTCCCAG<br>R: GTCGTATCCAGTGCAGGGT
**mmu-miR-27b-3p**	RT: GTCGTATCCAGTGCAGGGTCCGAGGTATTCGCACTGGATACGACAGGGATTC<br>F: AACAAGGTCCAGTTTTCCCAG<br>R: GTCGTATCCAGTGCAGGGT
**mmu_C/EBPα**	F: AGGTGCTGGAGTTGACCAGT<br>R: CAGCCTAGAGATCCAGCGAC
**mmu_PPARγ**	F: CAAGAATACCAAAGTGCGATCAA<br>R: GAGCTGGGTCTTTTCAGAATAATAAG
**mmu_FABP4**	F: AAGACAGCTCCTCCTCGAAGGTT<br>R: TGACCAAATCCCCATTTACGC
**mmu_U6**	RT: GTCGTATCCAGTGCAGGGTCCGAGGTATTCGCACTGGATACGACAAAATA<br>F: CTCGCTTCGGCAGCACA<br>R: AACGCTTCACGAATTTGCGT
**mmu_GAPDH**	F: TTGTTGCCATCAACGACCCC<br>R: GCCGTTGAATTTGCCGTGAG
**hsa-miR-145-5p**	RT: GTCGTATCCAGTGCAGGGTCCGAGGTATTCGCACTGGATACGACAGGGATTC<br>F: AACAAGGTCCAGTTTTCCCAG<br>R: GTCGTATCCAGTGCAGGGT
**hsa-miR-27b-3p**	RT: GTCGTATCCAGTGCAGGGTCCGAGGTATTCGCACTGGATACGACGCGGAACT<br>F: AACACGCTTCACGTGGCTA<br>R: GTCGTATCCAGTGCAGGGT
**hsa_C/EBPα**	F: AGGAGGATGAAGCCAAGCAGCT<br>R: AGTGCGCGATCTGGAACTGCAG
**hsa_PPARγ**	F: GGCTTCACATTCAGCAAACCTGG<br>R: AGCCTGCGAAAGCCTTTTGGTG
**hsa_FABP4**	F: ACCAGAGGATGATAAACTGGTGG<br>R: GCGAACTTCAGTCCAGGTGAAC
**hsa_PHB**	F: AAGCGGTGGAAGCCAAACAGGT<br>R: GCCAGTGAGTTGGCAATCAGCT
**hsa_LPL**	F: TGGAGGTACTTTTCAGCCAGGAT<br>R: TCGTGGGAGCACTTCACTAGCT
**hsa_IRS1**	F: GGAGTACATGAAGATGGACCTGG<br>R: CTGTTCGCATGTCAGCATAGC
**hsa_KLF4**	F: CATCTCAAGGCACACCTGCGAA<br>R: TCGGTCGCATTTTTGGCACTGG
**hsa_KLF5**	F: GGAGAAACGACGCATCCACTAC<br>R: GAACCTCCAGTCGCAGCCTTC
**hsa_U6**	RT: GTCGTATCCAGTGCAGGGTCCGAGGTATTCGCACTGGATACGACAAAATA<br>F: CTCGCTTCGGCAGCACA<br>R: AACGCTTCACGAATTTGCGT
**hsa_GAPDH**	F: GTCTCCTCTGACTTCAACAGCG<br>R: ACCACCCTGTTGCTGTAGCCAA

Supplementary References

1. Clevers H, Nusse R. Wnt/β-catenin signaling and disease. Cell. 2012; 149:1192–205. https://doi.org/10.1016/j.cell.2012.05.012 PMID:22682243

2. Hu HH, Cao G, Wu XQ, Vaziri ND, Zhao YY. Wnt signaling pathway in aging-related tissue fibrosis and therapies. Ageing Res Rev. 2020; 60:101063. https://doi.org/10.1016/j.arr.2020.101063 PMID:32272170

3. Teo YV, Rattanavirotkul N, Olova N, Salzano A, Quintanilla A, Tarrats N, Kiourtis C, Müller M, Green AR, Adams PD, Acosta JC, Bird TG, Kirschner K, et al. Notch Signaling Mediates Secondary Senescence. Cell Rep. 2019; 27:997–1007.e5. https://doi.org/10.1016/j.celrep.2019.03.104 PMID:31018144

4. Ong AL, Ramasamy TS. Role of Sirtuin1-p53 regulatory axis in aging, cancer and cellular reprogramming. Ageing Res Rev. 2018; 43:64–80. https://doi.org/10.1016/j.arr.2018.02.004 PMID:29476819

5. Principe DR, Diaz AM, Torres C, Mangan RJ, DeCant B, McKinney R, Tsao MS, Lowy A, Munshi HG, Jung B, Grippo PJ. TGFβ engages MEK/ERK to differentially regulate benign and malignant pancreas cell function. Oncogene. 2017; 36:4336–48. https://doi.org/10.1038/onc.2016.500 PMID:28368414

6. Wang H, Lu J, Stevens T, Roberts A, Mandel J, Avula R, Ma B, Wu Y, Wang J, Land CV, Finkel T, Vockley JE, Airik M, et al. Premature aging and reduced cancer incidence associated with near-complete body-wide Myc inactivation. Cell Rep. 2023; 42:112830. https://doi.org/10.1016/j.celrep.2023.112830 PMID:37481724

7. Mirabella AC, Foster BM, Bartke T. Chromatin deregulation in disease. Chromosoma. 2016; 125:75–93. https://doi.org/10.1007/s00412-015-0530-0 PMID:26188466

8. Katayama Y, Nishiyama M, Shoji H, Ohkawa Y, Kawamura A, Sato T, Suyama M, Takumi T, Miyakawa T, Nakayama KI. CHD8 haploinsufficiency results in autistic-like phenotypes in mice. Nature. 2016; 537:675–9. https://doi.org/10.1038/nature19357 PMID:27602517

9. Takeishi S, Matsumoto A, Onoyama I, Naka K, Hirao A, Nakayama KI. Ablation of Fbxw7 eliminates leukemia-initiating cells by preventing quiescence. Cancer Cell. 2013; 23:347–61. https://doi.org/10.1016/j.ccr.2013.01.026 PMID:23518349

10. Luo M, Li JF, Yang Q, Zhang K, Wang ZW, Zheng S, Zhou JJ. Stem cell quiescence and its clinical relevance. World J Stem Cells. 2020; 12:1307–26. https://doi.org/10.4252/wjsc.v12.i11.1307 PMID:33312400

11. Guo Y, Peng X, Cheng R, Chen H, Luo X. Long non-coding RNA-X-inactive specific transcript inhibits cell viability, and induces apoptosis through the microRNA-30c-5p/Bcl2-like protein 11 signaling axis in human granulosa-like tumor cells. Bioengineered. 2022; 13:14107–17. https://doi.org/10.1080/21655979.2022.2080366 PMID:35730492

12. Ortega-Molina A, Serrano M. PTEN in cancer, metabolism, and aging. Trends Endocrinol Metab. 2013; 24:184–9. https://doi.org/10.1016/j.tem.2012.11.002 PMID:23245767

13. Dupret B, Völkel P, Le Bourhis X, Angrand PO. The Polycomb Group Protein Pcgf1 Is Dispensable in Zebrafish but Involved in Early Growth and Aging. PLoS One. 2016; 11:e0158700. https://doi.org/10.1371/journal.pone.0158700 PMID:27442247

14. Eberhardt N, Kaur R, Das D, Amadori L, Sajja S, Bresciani J, Gildea M, Fernandez D, Rockman CB, Maldonado T. Zeb2 Regulates Senescence And Cytotoxic Phenotype In Atherosclerotic Plaque CD8 T Cells. Arteriosclerosis, Thrombosis, and Vascular Biology. 2024; 44(Suppl_1):A1030–A1030.

15. Li R, Chen B, Kubota A, Hanna A, Humeres C, Hernandez SC, Liu Y, Ma R, Tuleta I, Huang S, Venugopal H, Zhu F, Su K, et al. Protective effects of macrophage-specific integrin α5 in myocardial infarction are associated with accentuated angiogenesis. Nat Commun. 2023; 14:7555. https://doi.org/10.1038/s41467-023-43369-x PMID:37985764

16. Borghesan M, O'Loghlen A. Integrins in senescence and aging. Cell Cycle. 2017; 16:909–10. https://doi.org/10.1080/15384101.2017.1316573 PMID:28459356

17. Deng G, Zhang L, Wang C, Wang S, Xu J, Dong J, Kang Q, Zhai X, Zhao Y, Shan Z. AGEs-RAGE axis causes endothelial-to-mesenchymal transition in early calcific aortic valve disease via TGF-β1 and BMPR2 signaling. Exp Gerontol. 2020; 141:111088.

https://doi.org/10.1016/j.exger.2020.111088 PMID:32911032

18. Olkkonen VM. The emerging roles of OSBP-related proteins in cancer: Impacts through phosphoinositide metabolism and protein-protein interactions. Biochem Pharmacol. 2022; 196:114455. https://doi.org/10.1016/j.bcp.2021.114455 PMID:33556339

19. Zhu R, Ji X, Wu X, Chen J, Li X, Jiang H, Fu H, Wang H, Lin Z, Tang X, Sun S, Li Q, Wang B, Chen H. Melatonin antagonizes ovarian aging via YTHDF2-MAPK-NF-κB pathway. Genes Dis. 2020; 9:494–509. https://doi.org/10.1016/j.gendis.2020.08.005 PMID:35224163

20. Zhao Y, Liu YS. Longevity Factor FOXO3: A Key Regulator in Aging-Related Vascular Diseases. Front Cardiovasc Med. 2021; 8:778674. https://doi.org/10.3389/fcvm.2021.778674 PMID:35004893

21. Deng A, Ma L, Zhou X, Wang X, Wang S, Chen X. FoxO3 transcription factor promotes autophagy after oxidative stress injury in HT22 cells. Can J Physiol Pharmacol. 2021; 99:627–34. https://doi.org/10.1139/cjpp-2020-0448 PMID:33237807

22. Xie J, Ruan S, Zhu Z, Wang M, Cao Y, Ou M, Yu P, Shi J. Database mining analysis revealed the role of the putative H^+/sugar transporter solute carrier family 45 in skin cutaneous melanoma. Channels (Austin). 2021; 15:496–506. https://doi.org/10.1080/19336950.2021.1956226 PMID:34334114

23. Tu Z, Aird KM, Zhang R. Chromatin remodeling, BRCA1, SAHF and cellular senescence. Cell Cycle. 2013; 12:1653–4. https://doi.org/10.4161/cc.24986 PMID:23673322

24. Kwon J, Lee D, Lee SA. BAP1 as a guardian of genome stability: implications in human cancer. Exp Mol Med. 2023; 55:745–54. https://doi.org/10.1038/s12276-023-00979-1 PMID:37009801

25. Huang YZ, Zang M, Xiong WC, Luo Z, Mei L. Erbin suppresses the MAP kinase pathway. J Biol Chem. 2003; 278:1108–14. https://doi.org/10.1074/jbc.M205413200 PMID:12379659

26. Ho E, Irvine T, Vilk GJ, Lajoie G, Ravichandran KS, D'Souza SJ, Dagnino L. Integrin-linked kinase interactions with ELMO2 modulate cell polarity. Mol Biol Cell. 2009; 20:3033–43. https://doi.org/10.1091/mbc.e09-01-0050 PMID:19439446

27. Hutter D, Yo Y, Chen W, Liu P, Holbrook NJ, Roth GS, Liu Y. Age-related decline in Ras/ERK mitogen-activated protein kinase cascade is linked to a reduced association between Shc and EGF receptor. J Gerontol A Biol Sci Med Sci. 2000; 55:B125–34. https://doi.org/10.1093/gerona/55.3.b125 PMID:10795716

28. Xu X, Jin T. ELMO proteins transduce G protein-coupled receptor signal to control reorganization of actin cytoskeleton in chemotaxis of eukaryotic cells. Small GTPases. 2019; 10:271–9. https://doi.org/10.1080/21541248.2017.1318816 PMID:28641070

29. Todd AG, Shaw DJ, Morse R, Stebbings H, Young PJ. SMN and the Gemin proteins form sub-complexes that localise to both stationary and dynamic neurite granules. Biochem Biophys Res Commun. 2010; 394:211–6. https://doi.org/10.1016/j.bbrc.2010.02.158 PMID:20188701

30. Goldman RD, Shumaker DK, Erdos MR, Eriksson M, Goldman AE, Gordon LB, Gruenbaum Y, Khuon S, Mendez M, Varga R, Collins FS. Accumulation of mutant lamin A causes progressive changes in nuclear architecture in Hutchinson-Gilford progeria syndrome. Proc Natl Acad Sci USA. 2004; 101:8963–8. https://doi.org/10.1073/pnas.0402943101 PMID:15184648

31. Cong N, Du P, Zhang A, Shen F, Su J, Pu P, Wang T, Zjang J, Kang C, Zhang Q. Downregulated microRNA-200a promotes EMT and tumor growth through the wnt/β-catenin pathway by targeting the E-cadherin repressors ZEB1/ZEB2 in gastric adenocarcinoma. Oncol Rep. 2013; 29:1579–87. https://doi.org/10.3892/or.2013.2267 PMID:23381389

32. Wang J, Farkas C, Benyoucef A, Carmichael C, Haigh K, Wong N, Huylebroeck D, Stemmler MP, Brabletz S, Brabletz T, Nefzger CM, Goossens S, Berx G, et al. Interplay between the EMT transcription factors ZEB1 and ZEB2 regulates hematopoietic stem and progenitor cell differentiation and hematopoietic lineage fidelity. PLoS Biol. 2021; 19:e3001394. https://doi.org/10.1371/journal.pbio.3001394 PMID:34550965

33. Ramagoma RB, Makgoo L, Mbita Z. KLHL20 and its role in cell homeostasis: A new perspective and therapeutic potential. Life Sci. 2024; 357:123041. https://doi.org/10.1016/j.lfs.2024.123041 PMID:39233199

34. Koga H, Kaushik S, Cuervo AM. Protein homeostasis and aging: The importance of exquisite quality control. Ageing Res Rev. 2011; 10:205–15.

https://doi.org/10.1016/j.arr.2010.02.001 PMID:20152936

35. Zhen Y, Liu Z, Yang H, Yu X, Wu Q, Hua S, Long X, Jiang Q, Song Y, Cheng C, Wang H, Zhao M, Fu Q, et al. Tumor suppressor PDCD4 modulates miR-184-mediated direct suppression of C-MYC and BCL2 blocking cell growth and survival in nasopharyngeal carcinoma. Cell Death Dis. 2013; 4:e872. https://doi.org/10.1038/cddis.2013.376 PMID:24157866

36. Muradian K, Schachtschabel DO. The role of apoptosis in aging and age-related disease: update. Z Gerontol Geriatr. 2001; 34:441–6. https://doi.org/10.1007/s003910170015 PMID:11828881

37. Bridger JM, Kill IR. Aging of Hutchinson-Gilford progeria syndrome fibroblasts is characterised by hyperproliferation and increased apoptosis. Exp Gerontol. 2004; 39:717–24. https://doi.org/10.1016/j.exger.2004.02.002 PMID:15130666

38. Colucci-D'Amato L, Speranza L, Volpicelli F. Neurotrophic Factor BDNF, Physiological Functions and Therapeutic Potential in Depression, Neurodegeneration and Brain Cancer. Int J Mol Sci. 2020; 21:7777. https://doi.org/10.3390/ijms21207777 PMID:33096634

39. Hyatt DC, Ceresa BP. Cellular localization of the activated EGFR determines its effect on cell growth in MDA-MB-468 cells. Exp Cell Res. 2008; 314:3415–25. https://doi.org/10.1016/j.yexcr.2008.08.020 PMID:18817771

40. Geiger JA, Carvalho L, Campos I, Santos AC, Jacinto A. Hole-in-one mutant phenotypes link EGFR/ERK signaling to epithelial tissue repair in Drosophila. PLoS One. 2011; 6:e28349. https://doi.org/10.1371/journal.pone.0028349 PMID:22140578

41. Ge M, Sun L, Wang D, Hei C, Huang T, Xu Z, Shuai Q. Enhancement of therapeutic potential of mesenchymal stem cell by IGF-1 delivery in PLGA microspheres for tissue regeneration. Regen Ther. 2024; 27:112–19. https://doi.org/10.1016/j.reth.2024.03.004 PMID:38550913

42. Dharmawardana PG, Peruzzi B, Giubellino A, Burke TR Jr, Bottaro DP. Molecular targeting of growth factor receptor-bound 2 (Grb2) as an anti-cancer strategy. Anticancer Drugs. 2006; 17:13–20. https://doi.org/10.1097/01.cad.0000185180.72604.ac PMID:16317285

43. Sekiyama Y, Suzuki H, Tsukahara T. Functional gene expression analysis of tissue-specific isoforms of Mef2c. Cell Mol Neurobiol. 2012; 32:129–39. https://doi.org/10.1007/s10571-011-9743-9 PMID:21842419

44. Bano D, Piazzesi A, Salomoni P, Nicotera P. The histone variant H3.3 claims its place in the crowded scene of epigenetics. Aging (Albany NY). 2017; 9:602–14. https://doi.org/10.18632/aging.101194 PMID:28284043

45. Duarte LF, Young AR, Wang Z, Wu HA, Panda T, Kou Y, Kapoor A, Hasson D, Mills NR, Ma'ayan A, Narita M, Bernstein E. Histone H3.3 and its proteolytically processed form drive a cellular senescence programme. Nat Commun. 2014; 5:5210. https://doi.org/10.1038/ncomms6210 PMID:25394905

46. Saidak Z, Le Henaff C, Azzi S, Marty C, Marie PJ. Low-dose PTH increases osteoblast activity via decreased Mef2c/Sost in senescent osteopenic mice. J Endocrinol. 2014; 223:25–33. https://doi.org/10.1530/JOE-14-0249 PMID:25056116

47. Domínguez-Kelly R, Martín Y, Koundrioukoff S, Tanenbaum ME, Smits VA, Medema RH, Debatisse M, Freire R. Wee1 controls genomic stability during replication by regulating the Mus81-Eme1 endonuclease. J Cell Biol. 2011; 194:567–79. https://doi.org/10.1083/jcb.201101047 PMID:21859861

48. Gheghiani L, Wang L, Zhang Y, Moore XT, Zhang J, Smith SC, Tian Y, Wang L, Turner K, Jackson-Cook CK, Mukhopadhyay ND, Fu Z. PLK1 Induces Chromosomal Instability and Overrides Cell-Cycle Checkpoints to Drive Tumorigenesis. Cancer Res. 2021; 81:1293–307. https://doi.org/10.1158/0008-5472.CAN-20-1377 PMID:33376114

49. Fujise K, Zhang D, Liu J, Yeh ET. Regulation of apoptosis and cell cycle progression by MCL1. Differential role of proliferating cell nuclear antigen. J Biol Chem. 2000; 275:39458–65. https://doi.org/10.1074/jbc.M006626200 PMID:10978339

50. Tazawa H, Tsuchiya N, Izumiya M, Nakagama H. Tumor-suppressive miR-34a induces senescence-like growth arrest through modulation of the E2F pathway in human colon cancer cells. Proc Natl Acad Sci USA. 2007; 104:15472–7. https://doi.org/10.1073/pnas.0707351104 PMID:17875987

51. Sun F, Fu H, Liu Q, Tie Y, Zhu J, Xing R, Sun Z, Zheng X. Downregulation of CCND1 and CDK6 by miR-34a induces cell cycle arrest. FEBS Lett. 2008; 582:1564–8.

https://doi.org/10.1016/j.febslet.2008.03.057
PMID:18406353

52. DeNicola GM, Tuveson DA. RAS in cellular transformation and senescence. Eur J Cancer. 2009; 45 Suppl 1:211–6.
https://doi.org/10.1016/S0959-8049(09)70036-X
PMID:19775620

53. Murata A, Hayashi S. Notch-Mediated Cell Adhesion. Biology (Basel). 2016; 5:5.
https://doi.org/10.3390/biology5010005
PMID:26784245

54. Hong D, Zhang X, Li R, Yu J, Lou Y, He Q, Li X, Xu D, Lv P, Lin J, Chen Y. Deletion of TMEM268 inhibits growth of gastric cancer cells by downregulating the ITGB4 signaling pathway. Cell Death Differ. 2019; 26:1453–66.
https://doi.org/10.1038/s41418-018-0223-3
PMID:30361615
